# Evolution and the ultimatum game: An agent-based model with interbirth intervals and population structure

**DOI:** 10.1371/journal.pcbi.1014387

**Published:** 2026-06-16

**Authors:** Jeffrey C. Schank, Matt L. Miller

**Affiliations:** 1 Department of Psychology, University of California, Davis, California, United States of America; 2 Department of Psychology, Oakland University, Rochester, Michigan, United States of America; Interdisciplinary Transformation University IT:U, AUSTRIA

## Abstract

The ultimatum game (UG) is widely used to study mutually beneficial exchanges, fairness, and prosocial behavior across different societies. However, human behavior in UG experiments does not align with the game-theoretical prediction that proposers should offer the least positive amount and responders should accept such offers. Instead, proposers make generous offers that are greater than the minimum responders are willing to accept, resulting in generous offers with wide offer-acceptance gaps. Numerous evolutionary models of the UG have been created and studied to explain human behavior, particularly generous offers made in UG experiments. These models have recently faced criticism for lacking biological realism and not adequately explaining the data. Here, we present an agent-based model inspired by our hunter-gatherer ancestors and with a biologically more realistic selection process. We assume that (1) agents exist in group-structured and group-clustered populations, where reproduction (2) depends on resource accumulation, but (3) is limited by interbirth intervals. We ran simulations to assess whether this biologically more realistic model evolves patterns of behavior consistent with patterns in the data from meta-analyses of human behavior in the UG. For the proposed model, we show that generous offers robustly evolve, as well as the difficult-to-explain offer-acceptance gaps, only in group-structured populations with interbirth intervals. We demonstrate that these results are robust and may help explain variation in data across societies. We discuss how interbirth intervals interact with group structure to modulate offer and rejection costs, favoring the evolution of generous offers, offer-acceptance gaps, and other patterns in the data on human behavior in the UG. We also discuss why weak selection and/or high mutation rate models cannot explain all the patterns in UG experimental data. We discuss biological realism and conclude that group structure and interbirth intervals may be essential for explaining prosocial behavior across societies.

## Author summary

We used an agent-based model to investigate why people consistently make generous offers in the ultimatum game, contradicting classic game-theoretical predictions that proposers should offer the minimum possible and responders should accept any positive offer. Previous evolutionary models attempting to explain this generosity have been criticized for being biologically unrealistic. In response, our agent-based model reflects early human evolutionary conditions, incorporating group-structured populations, reproduction based on resource accumulation, and natural limitations on interbirth intervals (birth frequency). Our simulations show that generous offers and significant gaps between what is offered and what would be accepted evolve under these conditions, aligning with patterns found in real-world experimental data from different societies. Crucially, our model reveals that group structure and limits on the rate of reproduction interact to favor the evolution of generosity. We argue that these features are necessary for explaining cross-cultural variations in generosity, outperforming less realistic models relying only on weak selection or high mutation rates.

## Introduction

In its simplest form, the ultimatum game (UG) involves two people bargaining over a resource. A proposer offers a portion of the resource to a responder. If the responder accepts, the resource is divided as proposed, but if rejected, neither the proposer nor the responder receives anything. Nash equilibria exist when an acceptance threshold (AT), which is the minimum offer a responder is willing to accept, matches the amount the proposer is willing to offer. For example, if a responder will only accept half or more of a resource, and the proposer offers half, then one Nash equilibrium is a 50–50 split of the resource. Assuming individuals aim to maximize their payoffs during bargaining, the subgame-perfect Nash equilibrium can be determined through backward induction: the proposer should make the smallest non-zero offer, which the responder should accept as better than nothing [1,2].

The UG is widely used to study mutually beneficial transactions, fairness, and prosocial behavior across different societies. There have been many cross-cultural studies [3] and several meta-analyses [4–7] of the UG. However, experimental studies across societies consistently fail to find that people behave as predicted by the subgame-perfect Nash equilibrium or even that they use any Nash equilibrium strategies when playing the UG [3,4,6,7].

**Fig 1:**
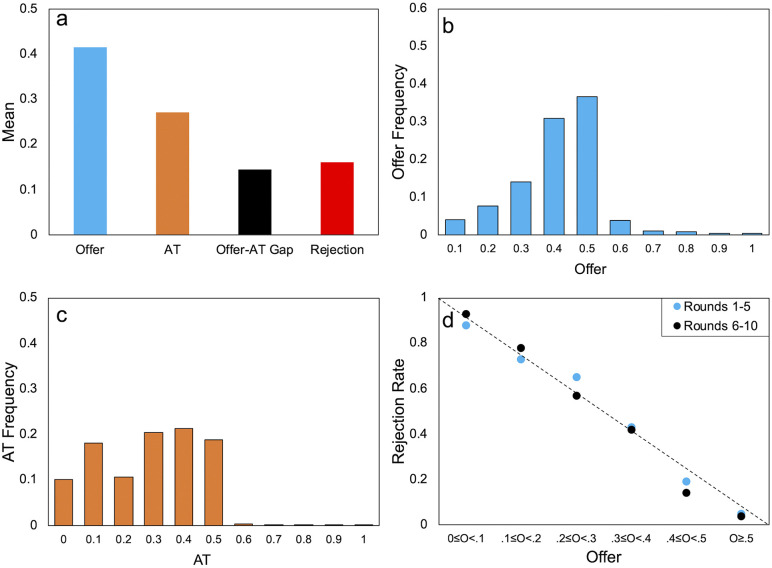
Empirical data from ultimatum game experiments. Meta-analysis mean offers, mean ATs, offer-AT gapa, and rejections **(a)**. Offer distributions across studies **(b)**. AT distribution from strategy method studies **(c)**. Relative rejection rates of responders with the dashed line indicating a linear decline from 1.0 for offers of 0.0 to 0.0 for offers ≥ 0.5 **(d)**. See S1 Text for sources of these data.

Data from several meta-analyses of the UG are summarized in Fig 1 (see S1 Text for an explanation of the data depicted in Fig 1). Mean offers (0.415) are well above those predicted by the subgame-perfect Nash equilibrium, and mean ATs (0.271) are well below mean offers (Fig 1a). If people used Nash equilibrium strategies, mean offers and ATs should be close and not differ by more than 0.144. This wide offer-AT gap is puzzling because it allows potential exploitation by stingier offer strategies and, therefore, theoretically, should not exist. Instead, we should find Nash equilibria in the data close to the subgame-perfect Nash equilibrium. Despite this wide offer-AT gap, offers are still rejected (0.16; Fig 1a). A distribution of offers pooled from 97 studies is plotted in Fig 1b [6], illustrating that the most common offer is 0.5. Indeed, less than 4% of offers fall within the subgame-perfect Nash equilibrium prediction for offers (0.1 or less). A distribution of ATs from UGs using the strategy method (e.g., see [8,9]) is illustrated in Fig 1c [10]. Finally, Fig 1d plots relative rejection rates of offers within ranges incrementing by 0.1 [5]. If players used subgame-perfect Nash equilibrium strategies, relative rejection rates would not fall linearly but would plummet to zero when offers are above the lowest range. In short, the data illustrated in Fig 1 empirically demonstrate that people do not behave as theoretically predicted when playing the UG. Players do not use Nash equilibrium strategies and mean offers, and ATs are not even close to those predicted by the subgame-perfect Nash equilibrium. In short, proposers often make generous offers, responders are willing to accept much lower offers, and there is significant variability among individuals in what they offer and accept. Additionally, rejection rates decrease linearly from very high for the lowest offers to very low for offers of half or more of a resource.

Generous mean offers repeatedly found in these experiments have motivated the creation of many evolutionary models of the UG (Table 1). Debove et al. [11] reviewed and classified many of these models into six categories, to which we added a kin-selection category (Table 1). Most of these models aim to show how the mean offers higher than predicted by the subgame-perfect equilibrium could evolve, but they do not typically address any of the other patterns of behavior in the UG (Fig 1).

**Table 1:** Model categories and examples. Definitions follow Debove et al. [11] with our addition of a kin-selection category. Examples from Debove et al. with the addition of models published since following the same criteria.

Model categories	Model Description
**Alternating role** Rubinstein [12]; Hoel [13] **Reputation** Nowak et al. [14]; Chiang [15]; André and Baumard [16]; Zhang, et al. [17]; Deng et al. [18]; Deng et al. [19] **Noise-based/Mutation Selection** Roth and Erev [20]; Binmore and Samuelson [21]; Gale et al. [22]; Harms [23]; Zollman [24]; Rand et al. [25]; Skyrms [26]; Santos et al. [27]; da Silva et al. [28,29]; Zhang et al. [30]; Wang et al. [31]; Akdeniz and van Veelen [10,32]; Li et al. [33]; Arioli et al. [34]; Vanderschraaf [35]; Wu et al. [36] **Spite** Huck and Oechssler [37]; Barclay and Stoller [38]; Forber and Smead [39] **Spatial population structure** Page et al. [40]; Killingback and Studer [41]; Alexander [42]; Iranzo et al. [43]; Ye et al. [44]; Zhang et al. [45]; Alves et al. [46]; Chen et al. [47]; Deng et al. [48]; Zhao et al. [49]; Chen et al. [50]; Cimpeanu et al. [51]; Deng et al. [52,53]; Zheng et al. [54–56]; Li et al. [57]; Wang et al. [58]; Charcon and Monteiro [59] **Empathy-based** Page and Nowak [60]; Sánchez and Cuesta [61]; Zhang et al. [62]; Gao et al. [63] **Kin-selection** Hintze and Hertwig [64]; Wild et al. [65]	Mathematical models of alternating or random alternating roles of a proposer and a responder Population models in which reputation influences the offers made or partners chosen to play Population models with randomness or mutation in offers and ATs evolved using learning models or selection models Models that inflict costs by rejecting low offers Models in which players are arranged on a square grid ring, network, etc. Models assuming players are empathetic, e.g., assuming *p* = *q* Models assuming players make decisions based on relatedness

Of the models reviewed by Debove et al. [11], noise-based or what we will call mutation-selection models attempt to directly address the evolution of generous offers and, to a lesser extent, offer-AT gaps (e.g., Akdeniz and van Veelen [10,32]). These approaches generally find that if mutation rates are high and selection is weak, mean offers and offer-AT gaps can evolve but often differ from experimental data [11]. In these mutation-selection models, selection is modeled as proportional to the average payoff of strategies, and it is implemented using normalized exponential probability distributions. A problem with this approach is that it does not accurately reflect how biological mechanisms and constraints influence fitness (see Discussion). Debove et al. ([11] p. 250) highlighted the challenge of prioritizing parsimony over realism in modeling, concluding that “...parsimony often comes at a cost regarding the biological credibility of the model.” Furthermore, from a biological standpoint, a difficulty with high-mutation, weak-selection models is providing a rationale for why selection should be weak or mutation so high, especially for behaviors (i.e., resource division) that are essential for human fitness.

We agree with Debove et al. [11] that models of the UG should make better contact with biological and social reality. Here, we begin with the question: under what conditions does selection favor the evolution of generous offers and wide offer-AT gaps similar to those observed in empirical research (see Fig 1)? We assume that the plausibility of a model depends on its consistency or alignment with multiple patterns in the data from UG experiments, as depicted in Fig 1. We propose a new approach that models selection based on generic biosocial constraints that our hunter-gatherer ancestors faced. We assume that, like our hunter-gatherer ancestors, (1) agents exist in group-structured and group-clustered populations [66], (2) reproduction requires the accumulation of resources, but (3) the rate of reproduction is constrained by interbirth intervals (IBIs; [67]). Selection emerges from playing the UG under these three assumptions, which place this model under the categories of mutation-selection and spatial population structure (Table 1). We will use simple genetic models of inheritance here because our focus is on the importance of interbirth intervals and population structure. Future investigations could employ cultural evolution models and social learning, as we discuss in the Discussion section, but there are challenges in finding social learning models that align with the assumptions we make in this model.

We show that introducing IBIs has a negligible effect on the evolution of offers and ATs in large, well-mixed populations (i.e., compared to reproduction without IBIs). However, in group-structured populations where reproduction is constrained by IBIs, simulated mean offers, mean ATs, offer-AT gaps, offer and AT distributions, and relative rejection rates align broadly with observed patterns in the corresponding experimental data depicted in Fig 1. We discuss how generous offers and wide offer-AT gaps evolve because IBIs and group-structure modulate offer and rejection costs in the UG. We also discuss why neither weak selection nor high mutation rates can adequately explain data from UG experiments. We conclude by discussing the importance of more realistic selection models and alternative paths to models that may lead to truer theories [68].

## Methods and models

**Fig 2:**
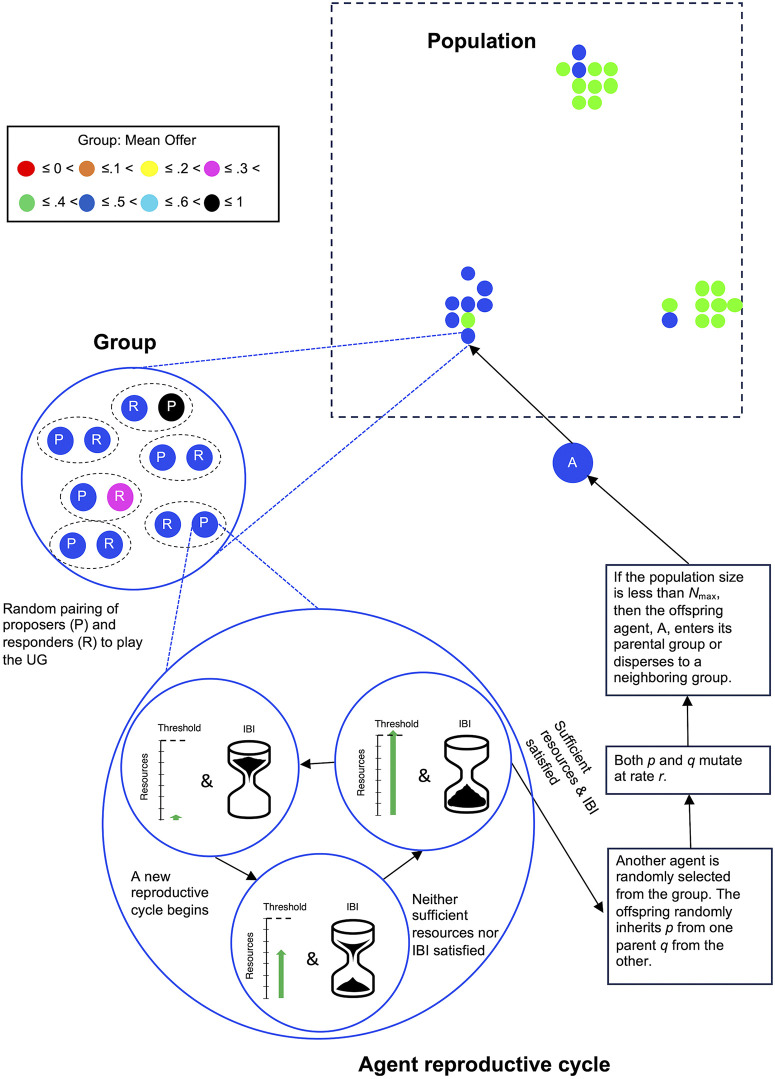
Model diagram of the levels represented in the model, including IBIs. At the top is a portion of the population from Fig 3. In the middle, an arbitrary group is blown up to show agents randomly assigned to play UGs. At the bottom is a depiction of an IBI with an offspring in the parental group.

The model spans multiple levels of organization consisting of populations containing clusters of groups, UGs, and agents (Fig 2). Within groups, agents are randomly paired to play a UG for resources at each time step. The UG in this context could be thought of as analogous to the stag hunt game [69]: one agent proposes a cooperative endeavor to obtain resources (e.g., as in a hunt for a stag) and offers a split of the resources, which, if accepted, is assumed to be successful, and the resources are divided as proposed; otherwise, both receive nothing. IBIs limit the rate of reproduction, which requires accumulating resources from playing UGs. We then systematically ran multiple sets of simulations to compare well-mixed versus structured populations with and without IBIs. We also ran simulations to assess the effects of mutation rate, dispersion, population density, sexual vs asexual reproduction, and population size.

### Populations and groups

**Fig 3:**
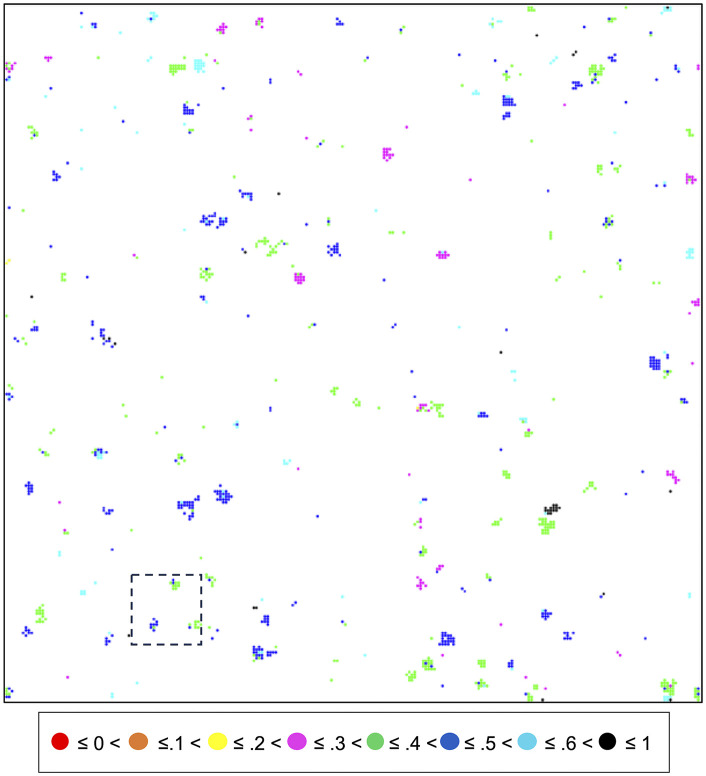
Example spatial distribution of groups. Parameters were *G*_max_ = 20, *N*_max_ = 10,000, *m* = 150, after 200 generations in 250 × 250 discrete space (see Table 2 for a description of these parameters). Groups are color-coded by the average offer of their members. The dashed rectangular region is the portion of the population represented in Fig 2.


Populations are either well-mixed (any agent can randomly play any other agent) or are group-structured (an agent can only randomly play agents from their own group). For group-structured populations, groups are located in a square, *s* × *s*, 2D discrete toroidal space (Fig 3). At the start of a simulation, the initial size of groups is *G*_max_/2, where *G*_max_ is the maximum group size, and the initial number of groups is 2*N*_max_/*G*_max_, where *N*_max_ is the maximum population size. The density *ρ* of groups in 2D space is the initial number of groups divided by the area of the space. Eq (1) defines the length of a side *s* of a square 2D space in terms of *N*_max_, *G*_max_, and *ρ* (i.e., the density of groups) to maintain a constant initial density across conditions:

$$\begin{array}{c} s=\text{round}\left(\text{sqrt}\left(\frac{{\text{2}N}_{\text{max}}}{{\rho G}_{\text{max}}}\right)\right) \end{array}$$ (1)

When a group reaches *G*_max_, a fission event is triggered. In a group fission event, each agent in the parent group has a 0.5 probability of moving to the offspring group using a rejection method that requires both groups to have at least two members. The offspring group either disperses globally (i.e., a random empty location in 2D space) with probability *g* or locally (i.e., a random empty location in its parental Moore neighborhood with a radius of 1) with probability 1.0 – *g* to another location in 2D space. For local dispersion, there may be no empty cells in the Moore neighborhood of the parent group; in such cases, the parent group does not fission, but another fission event is triggered on the next time step if the group size is greater than or equal to its maximum size, *G*_max_. When a group has fewer than two members, the remaining member disperses to the nearest group (described below), and the empty group is removed from the population. This assumption guarantees at least two members in a group to play the UG and for sexual reproduction. This fission process allows clusters to form in space, but also allows groups to be dispersed in space (see Fig 3 from an example simulated group structure).

### Agents

#### 1. UGs, resources, and IBIs.

Agents play UGs to accumulate resources to reproduce. The *x* resources bargained for are drawn from a truncated Gaussian distribution [70] with mean *µ*, standard deviation *σ*_*µ*_, lower bound *a* = 0, and upper bound *b =* 2*µ*. The proposer offers a portion, *px,* to a responder, and if *px* ≥ *qx*, the proposer keeps (1 – *p*)*x*, and the responder receives *px*; otherwise, both receive nothing.

On each time step, an agent participates in one UG with a randomly selected member of its group (in group-structured populations) or from a single group of all agents constituting the whole population (for well-mixed populations). An agent’s role as proposer or responder is determined randomly with a probability of 0.5. The assumption that each agent participates in one game per time step ensures that each agent has an equal opportunity to obtain resources on each time step. However, groups and populations can contain even or odd numbers of agents, which is an especially important problem for group-structured populations. If an agent does not play in an odd group, this would violate the assumption of equal opportunity to obtain resources and change the expected resources for agents in odd groups. Thus, for the odd agent not paired, we randomly assigned it the role of proposer or responder and randomly selected another agent from its group to play the opposing role. The odd agent keeps its share if there is an acceptance, while the randomly selected partner keeps nothing, whether or not there is an acceptance, because it has already participated in a UG. Because this is a novel approach, we investigated this assumption against alternative rules for handling odd agents in the Results section.

An IBI is the period of time between two consecutive births [71]. For iteroparous species such as humans, which reproduce multiple times during their lifespan, IBIs are one of three determinants of reproductive fitness, together with offspring mortality and lifespan [72]. In present-day human natural fertility populations, IBIs are approximately three years in length with considerable variation [73], but IBIs less than three years substantially increase infant mortality [74]. The minimum duration of an IBI will vary with the environment and society, but has three phases: (i) gestation, (ii) postpartum anovulation characterized by lactation and infant care suppressing reproductive function, and (iii) cycling. The first phase is the least variable component, postpartum anovulation is the most variable, and all phases are sensitive to resource availability [75]. In this model, we assume that the minimum IBI required to produce viable offspring is *m* time steps and that the *m* time steps in an IBI can be interpreted, for example, as days, weeks, or months and is the minimum number of UGs played during an IBI. For instance, if the minimum IBI for agents is approximately three years and a UG is played approximately weekly, then *m* = 150 could represent a three-year IBI. If too few resources are accumulated, producing viable offspring may take longer than *m* time steps, but *m* is the minimum time required. However, because we do not know the time interval between UGs, it is critical to investigate a wide range of possible values. Here we simulated *m* = 25, 50, 100, 150, 200, 300, 400, and 500. In short, we assume that (i) a minimum IBI is of length *m*, (ii) at least *m* UGs are played during an IBI, and (iii) the accumulation of *θ* resources is required to reproduce.

If all offers in a group or population are the *same* and accepted, the expected payoff per time step is *µ*/2 (i.e., one-half of the mean, *µ*, endowment resources bargained for), but if any offers are rejected, the expected payoff is less than *µ*/2. Thus, if a population has reached any Nash equilibrium (e.g., it could be the subgame-perfect or 50–50 Nash equilibrium), or all the offers are the same and acceptable to all, then the expected accumulation of resources, *θ*, is defined by Eq (2).


$$\begin{array}{c} \theta =m\frac{\mu }{\text{2}} \end{array}$$ (2)

Our characterization of the minimum IBI as *m* time steps recognizes that the actual number of time steps will vary with the outcome of UGs. While agents must accumulate at least *θ* resources during an IBI to reproduce, for agents constrained by IBIs, accumulating more resources before *m* time steps have passed is not sufficient to reproduce (e.g., consuming more food than needed does not shorten the minimum time required for gestation, infant care, and cycling), though we assume that excess resources are carried over to the next IBI as defined in Eq (3)


$$\begin{array}{c} U_{i,t}=U_{i,t-\text{1}}-\theta \end{array}$$ (3)

where *U*_*i,t*_ is the accumulated resources at time step *t* updated if a reproductive event (i.e., whether or not an offspring is successfully reproduced) is triggered and *θ* resources are subtracted.

A reproductive event for agents constrained by IBIs is triggered when condition (4) is true


$$\begin{array}{c} \tau_{i}\geq mU_{i,t}\geq \theta \end{array}$$ (4)

where *𝜏*_*i*_ is the number of time steps since the *i*th agent was reproduced or previously reproduced an agent. For agents unconstrained by IBIs, a reproductive event is triggered when condition (5) is true.


$$\begin{array}{c} U_{i,t}\geq \theta \end{array}$$ (5)

We assume a reproductive process in which an offspring agent successfully enters the population only if the current population size *N*_*t*_ < *N*_max_; otherwise, the offspring agent dies [67,76]. This condition holds for both well-mixed and group-structured populations. Thus, for an agent to successfully reproduce, a reproductive event must be triggered (i.e., a condition satisfying Eq (4) for agents with IBIs or Eq (5) for agents without IBIs) and population size *N*_*t*_ < *N*_max_. For group-structured populations, offspring agents disperse to a non-parental group (if possible) with probability *d* and remain in their parental group with probability (1 – *d*). The development of agents is not included in this model, and thus, offspring agents are immediately adults, an implicit assumption in most evolutionary UG models.

#### 2. Reproduction.

A more biologically realistic approach to reproduction might involve male and female agents who pass on heritable strategies to their offspring. Experimental research has shown that additive genetic effects explain 42% of responder behavior in the UG [77], but it remains unclear how social learning, evolved psychological mechanisms, and cultural transmission also influence the transmission of strategies or preferences. Although creating more realistic mating and reproductive systems is a future goal, since our focus is on developing a biologically more accurate selection model, understanding the role of IBIs is crucial. At this stage of model development, adding more realism would complicate the analysis and interpretation of the simulation, so we present the current model as an initial step.

Humans reproduce sexually, and there are two advantages of sexual reproduction, as opposed to asexual reproduction, that may be important in the context of the UG. First, sexual reproduction, by recombining strategies, may allow agents to better adapt to their environment [78]. Second, sexual reproduction can also help eliminate bad strategies (i.e., bad offer-AT combinations) by recombining them and allowing selection to remove them [78]. To capture these two benefits of sexual reproduction without modeling human mating systems or the likely polygenic determination of human behavior in the UG, we assume a simple sexual system of reproduction that allows offers and ATs to be randomly shuffled in a group or the whole population to implement Crow’s [78] two advantages of sexual reproduction (i.e., recombining offers and ATs to better adapt to a changing social environment and combine bad strategies to eliminate them). While biologically unrealistic in many respects, the random shuffling of offers and ATs is a step towards more realism than the asexual approaches often used [10].

To compare the effects of sexual versus asexual reproduction in this model, we also modeled asexual reproduction. For sexual and asexual reproduction, an agent *i*’s haploid genome consists of an offer portion, 0 ≤ *p*_*i*_ ≤ 1, and an AT, 0 ≤ *q*_*i*_ ≤ 1, which an offspring inherits from their parents in sexual reproduction and from one parent in asexual reproduction. For simulations with sexual reproduction only, an offspring inherits either *p* or *q* randomly from the parent that meets either condition (4) or (5), and the other *p* or *q* from a randomly selected agent from their group or the whole population in well-mixed populations. For simulations with asexual reproduction only, an offspring inherits both *p* and *q* from a single parent. The *p* and *q* loci of the offspring’s chromosome each mutate independently with probability *r* for both forms of reproduction.

#### 3. Lifespan.

Agents have a lifespan, a multiple of the expected number of IBIs they will have. More specifically, agents have a mean lifespan, *L*, which is a multiple of *m*: *L* = *δm*, where δ = 4 is the expected number of IBIs during a lifespan. The lifespan of an agent is a major factor in the use of computational resources, and preliminary simulations indicated that increasing δ did not affect the results. The lifespan, *L*_*i*_, of each agent *i* was randomly drawn from a Gaussian distribution with standard deviation, *σ*_*L*_ = 0.1*L* (e.g., if *m* = 150, *L* = 600, and *σ*_*L*_ = 60).

#### 4. Dispersion.

In group-structured populations, dispersion occurs in two contexts. First, agents disperse to another group with probability *d* at birth. When agents disperse, they disperse to another group in the Moore neighborhood of their parental group, which implements a stepping-stone model of dispersion [79]. If there is no group in the Moore neighborhood of the parental group, the agent does not disperse (see Fig 3 for an example of the population structure that emerges in simulations). Second, when a group is reduced to a single agent, the agent disperses to the nearest group. If there is more than one group equally distant, a random equidistant group is selected. This assumption guarantees that groups always contain two or more agents.

### Simulations

We aimed to assess the empirical plausibility of this model by comparing patterns of simulation results to patterns in the experimental data on the UG (see Fig 1). To accomplish this aim, we systematically ran simulations to compare well-mixed versus structured populations with and without IBIs. We investigated the effects of varying IBIs, *m*, the maximum size of groups, G_max_, mutation rates, *r*, dispersion, *d*, group density, *ρ*, resource variation, *σμ*, and expected resource accumulation, *θ*, relative to IBIs, *m*. The parameters, variables, and initial conditions of simulations are listed in Table 2. Although the parameters and variables are intended to represent characteristics of hunter-gatherer populations, the values used were selected to illustrate some possibilities and demonstrate the plausibility of this modeling approach for explaining experimental data from UG experiments.

**Table 2:** Initial conditions, parameters, and variables.

Symbols	Values and interpretations
**Parameters** *N*_max_ *G*_max_ *ρ* *s* × *s* *g* *μ* *σ*_*μ*_ *a* *b* *θ* *δ* *m* *L* *σ*_*L*_ *r* *d* *α* **Variables** *p* *q* *N*_*t*_ *U*_*i,t*_ *x* *L*_*i*_ **Initial conditions** Random Zero *N*_0_ *U*_*i*,*t*=0_ *L*_*i*_	10,000, the maximum number of agents for most simulations Maximum group size: 20, 40, 60, 80, 100 0.016, 0.032, the density of groups 250 × 250, 177 × 177, 144 × 144, 125 × 125, 112 × 112, which correspond respectively to *G*_max_ = 20, 40, 60, 80, 100 0.1, the probability of global dispersion during group fission 10, mean endowment resources for a UG 0, 0.1*μ*, 0.25*μ*, 0.5*μ*, 0.75*μ* endowment resource standard deviations 0, lower bounds for the endowment resources for a UG 2*μ*, upper bounds for the endowment resources for a UG *mμ*/2 = *5m*, the quantity of resources required to reproduce 4, the maximum number IBIs in a lifespan 25, 50, 100, 150, 200, 300, 400, 500, the minimum number of time steps and number of UGs played during an IBI *δm*, mean agent lifespan for all simulations 0.1*L*, standard deviation computed for each simulation 0.01, mutation probability per locus for most simulations 0.1, offspring dispersal probability for most simulations Coefficient for changing *θ*, resource required for reproduction Offer strategy of an agent with the range [0, 0.1, 0.2,..., 1.0] AT of an agent with the range [0, 0.1, 0.2,..., 1.0] Number of agents in a population at time *t* The resources accumulated by agent *i* at time *t*; at birth Resource endowment for a UG Lifespan of an individual agent At the start of a simulation, *p* and *q* are uniform randomly assigned values from the range [0, 0.1, 0.2,..., 1.0] for all agents At the start of a simulation, *p* = 0 and *q* = 0 for all agents 10,000 at the start of a simulations, *t* = 0, for most simulations 0, starting resources At the start of a simulation, lifespans are uniform randomly assigned integers in the range 0 to *L*

While most simulations were run with sexual reproduction, we also compared some of these results with simulations using asexual reproduction. The nine simulation studies conducted to investigate the effects of group structure and IBIs are listed in Table 3.

**Table 3:** Simulation studies.

Studies	Initial conditions and parameters varied
**Study 1: Well-mixed populations** **Study 2: Group-structured populations** **Study 3: Mutation rates** **Study 4: Dispersion** **Study 5: Density** **Study 6: Asexual reproduction** **Study 7: Population size** **Study 8: Resource shortages and surpluses** **Study 9: Odd groups**	*d* = 0.1; *σ*_*μ*_ = 0.5*μ*; *r* = 0.01; *N*_max_ = 10,000; *ρ* = 0.016; Initial strategies: Random, Zero; *G*_max_ = *N*_max_; IBIs: with and without; *m* = 25, 50, 100, 150, 200, 300, 400, 500; Populations structure: Well-mixed *d* = 0.1; *σ*_*μ*_ = 0, 0.1*μ*, 0.25*μ*, 0.5*μ*, 0.75*μ*; *r* = 0.01; *N*_max_ = 10,000; *ρ* = 0.016; Initial strategies: Random, Zero; *G*_max_ =10, 20, 40, 60, 100; IBIs: with and without; *m* = 25, 50, 100, 150, 200, 300, 400, 500; Populations structure: Well-mixed, Group *d* = 0.1; *σ*_*μ*_ = 0.5*μ*; *r* = 0.001, 0.01, 0.1; *N*_max_ = 10,000; *ρ* = 0.016; Initial strategies: Random; *G*_max_ = 20, 40, 100; IBIs: with; *m* = 25, 50, 100, 150, 200, 300, 400, 500; Populations structure: well-mixed, group *d* = 0.1 0.2, 0.3, 0.4, 0.5; *σ*_*μ*_ = 0.5*μ*; *r* = 0.01; *N*_max_ = 10,000; *ρ* = 0.016; Initial strategies: Random; *G*_max_ = 20, 40, 100; IBIs: with; *m* = 25, 50, 100, 150, 200, 300, 400, 500; Populations structure: Group *d* = 0.1;*σ*_*μ*_ = 0.5*μ*; *r* = 0.01; *N*_max_ = 10,000; *ρ* = 0.016, 0.032; Initial strategies: Random; *G*_max_ =20, 40, 100; IBIs: with; *m* = 25, 50, 100, 150, 200, 300, 400, 500; Populations structure: Group Same as Study 2 but only with IBIs and asexual reproduction *d* = 0.1;*σ*_*μ*_ = 0.5*μ*; *r* = 0.01, *N*_max_ = 30,000; *ρ* = 0.016; Initial strategies: Random; *G*_max_ = 60; IBIs: with; *m* = 25, 50, 100, 150, 200, 300, 400, 500; Populations structure: Group *d* = 0.1; *σ*_*μ*_ = 0.5*μ*; *r* = 0.01; *N*_max_ = 10,000; *ρ* = 0.016; Initial strategies: Random; *G*_max_ = 20, 40, 60,100;; IBIs: with; *m* = 25, 50, 100, 150, 200, 300, 400, 500; Populations structure: Group; Resource shortage: *α* = 1.01, 1.02, 1.03, 1.04, 1.05, 1.06, 1.07, 1.08, 1.09, 1.1; Resource abundance: *α* = 0.99, 0.98, 0.97, 0.96, 0.95, 0.94, 0.93, 0.92, 0.91, 0.9 *d* = 0.1; *σ*_*μ*_ = 0.5*μ*; *r* = 0.01; *N*_max_ = 10,000; *ρ* = 0.016; Initial strategies: Random; *G*_max_ = 20, 40, 60,100;; IBIs: with; *m* = 25, 50, 100, 150, 200, 300, 400, 500; Populations structure: Group; Odd group rules: FGR, KR, NPR compared to HGR

The maximum population size, *N*_max_ = 10,000 (except for Study 7), was set so that populations would start with 200–1000 groups, thereby avoiding small-population effects at the group level. Values for maximum group size, *G*_max_, were selected to explore a range of group sizes, and the global dispersion *g* = 0.1 was selected to generate clusters of groups [66] as illustrated in Fig 2. Larger values of *g* reduce cluster size. The discrete space dimensions, *s* × *s*, were arbitrarily selected to be 250 × 250 for *G*_max_ = 20. The density *ρ* = 0.016 was then calculated based on 1000 initial groups, and using Eq (1), the dimensions of the spaces for other *G*_max_ were calculated. Dispersal rates among our early hunter-gatherer groups are unknown, so we set *d* = 0.1 for most simulations. We later show that the results are not sensitive to dispersal probabilities up to *d* = 0.5. Offer strategies, *p*, and ATs, *q*, were restricted to the range [0, 0.1, 0.2, …, 1.0] because increments of 10% of an endowment are commonly used in UG experiments (e.g., Henrich et al. [3]).

All group-structured population simulations began with groups randomly dispersed in 2D space and with *N*_0_ = *N*_max_ initial agents evenly distributed among groups so that each group contained *G*_max_/2 agents. For example, at the start of a simulation for *N*_max_ = 10,000, *G*_max_ = 20, 1000 groups were constructed with 10 agents each. Well-mixed population simulations also started with *N*_0_ = *N*_max_ agents in one group with *G*_max_ = *N*_0_ = *N*_max_, so the group never fissions nor is there dispersion. The dimensions of the 2D discrete spaces for group-structured populations were calculated, Eq (1), so that the initial density, *ρ*, of groups over the discrete locations in space was held constant (see Table 2; see Fig 3 for an example of a distribution of groups in a 2D space; see Fig A in S2 Text for descriptive statistics of group-structured populations).

Two approaches were investigated for the starting strategies of a population of agents: Random and Zero. For Random initial strategies, both *p* and *q* for all agents were randomly selected from the range [0, 0.1, 0.2, …, 1]. Random initial strategies are typical for mutation-selection models, suggesting this might be problematic because some agents will initially start making generous offers [11]. However, starting from *p* = *q* = 0 relies solely on the mutation rate to introduce variation and assumes all agents accept nothing at the start of simulations. Starting with Random initial strategies, variation is already present in the population for offers and ATs, and the evolutionary process sorts it out. We prefer Random initial strategies for introducing strategy variation at the start of simulations, but we also compare Random to Zero initial conditions in the first two simulation studies to assess any differences.

The lifespan of each agent at the start of a simulation was drawn from a uniform random distribution of integers in the range [0, *L*]. This allowed the initial generation to have lifespans uniformly distributed over the first generation for an initial burn-in period. Starting resources, $U_{i,t=\text{0}}$, for agents were set to 0.

All simulations ran for 1,000 generations, where a generation is *L* time steps. For example, if *m* = 25, then *L* = *δm* = 100, and simulations were run for 100,000 time steps. The longest simulations were *m* = 500 with *L* = *δm* = 2000, and simulations ran for 2,000,000 time steps. For each combination of parameter values, 10 simulations were run.

For each combination of parameter values, simulation data were recorded from the last 100 generations (preliminary simulations demonstrated that mean offers and ATs changed little after 500 generations), sampled every 10 generations, and averaged over 10 simulations in the set.

Rejection rates were also calculated every 10 generations for the last 100 generations of a simulation, but were calculated by randomly selecting 1000 agents from a population every 10 generations, randomly pairing them to play 500 UGs, and then counting the number of rejections. The choices of proposer and responder were independent of group membership, so for group-structured populations, agents were typically randomly paired from different groups. The mean rejection rate was the average across samples from a simulation and over the 10 simulations in a set. Relative rejection rates for all levels of offers were also calculated, and offers ≥ 0.5 were aggregated together.

The model, data collection, and recording procedures were written in Java using the Mason version 20 agent-based modeling library [80].

## Results

### Well-mixed populations

**Fig 4:**
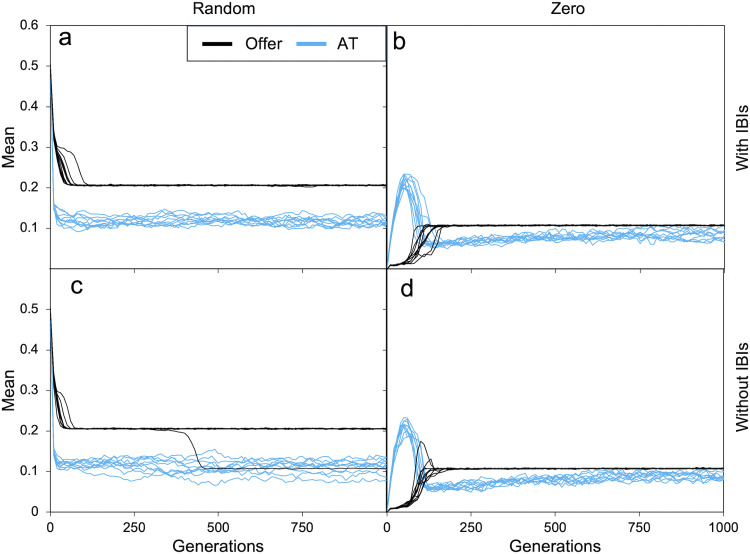
Evolutionary trajectories for individual simulations in well-mixed populations. Parameter values were *m* = 150 and *σ*_*µ*_ = 0.5*µ* with **(a, b)** and without **(c, d)** IBIs, and for Random and Zero initial conditions.

We initially ran simulations in unstructured populations (Study 1, Table 3) and found that IBIs had minimal or no impact on evolved offers and ATs (Fig 4). Because the minimum value of an offer, *p* > 0, is 0.1 (Table 2), if a population evolves the Nash equilibrium, evolved mean offers should approach 0.1. For the Random initial condition, mean offers evolved to a mean of approximately 0.2, except for one simulation without IBIs, which dropped to approximately 0.1 between 250 and 500 generations (Fig 4c). For the Zero starting condition, mean offers evolved to a mean of approximately 0.1, which approximated the Nash equilibrium of 0.1 with and without IBIs. Mean ATs evolved to slightly higher than 0.1 in Random and slightly lower than 0.1 in Zero initial conditions (Fig 4). Thus, well-mixed populations, with or without IBIs, evolved similar mean offers and ATs depending on Random or Zero initial conditions.

**Fig 5:**
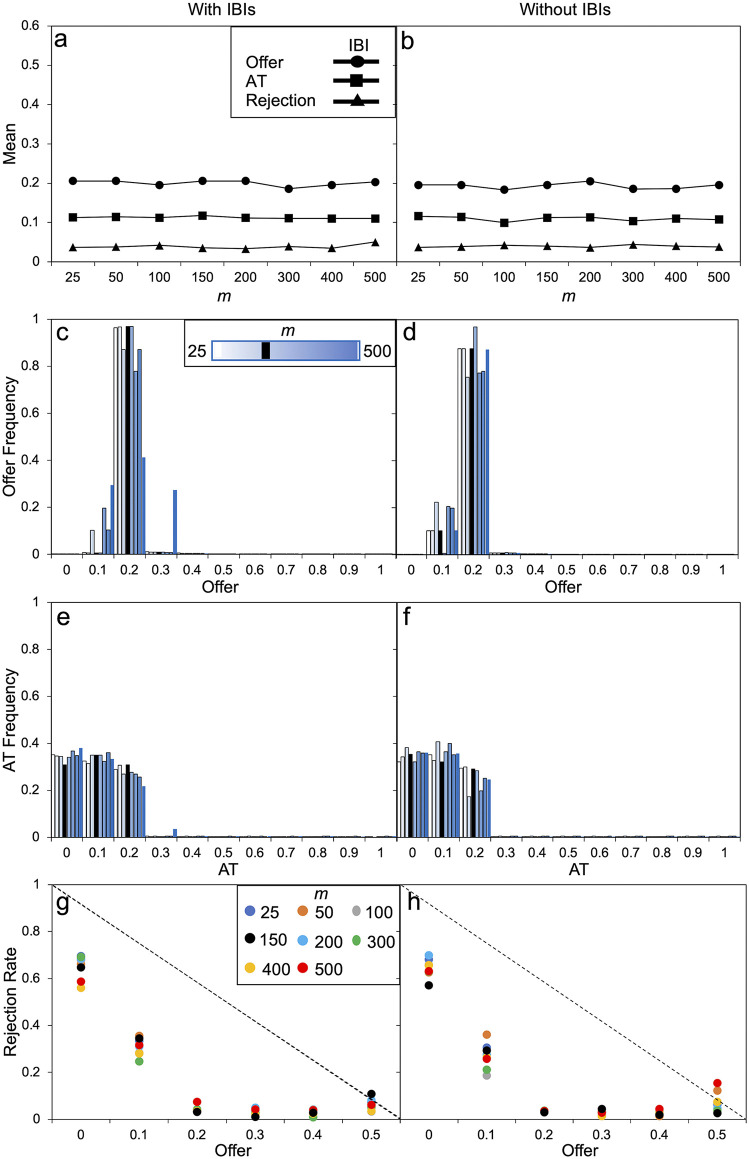
Simulation results for well-mixed populations with and without IBIs. Simulations started with Random initial conditions and *σ*_*µ*_ = 0.5*µ*. The top row plots mean offers, mean ATs, and mean rejection rates with **(a)** and without **(b)** IBIs. The middle rows are offer distributions for *m* = 25 to 500 (in shades of blue with *m* = 25 shaded white and *m* = 150 shaded black for reference) with **(c)** and without **(d)** IBIs, and AT distributions with **(e)** and without **(f)** IBIs. The bottom row illustrates relative rejection rates (dashed lines indicate a linear decline from 1.0 for offers of 0 to offers ≥ 0.5) with **(g)** and without **(h)** IBIs.

More generally, for the Random initial condition and varying *m*, mean offers evolved to approximately 0.2 with or without IBIs, and mean ATs evolved to close to 0.11 with or without IBIs with an offer-AT gap of 0.09 (Fig 5a-5b). Mean rejection frequencies were low (3% to 5%) with or without IBIs (Fig 5a-5b). The distributions of offers were similar with or without IBIs except for *m* = 500 in the IBI condition, where offers of 0.3 appear, as well as ATs at very low frequencies (Fig 5e-5f). Relative rejection rates dropped to low levels when offers reached 0.2 but increased slightly for offers greater than or equal to 0.5 with or without IBIs (Fig 5g-5h). Corresponding results (not depicted) for the Zero initial condition evolved to approximately 0.1, and mean ATs evolved between 0.08 and 0.09 with or without IBIs. Therefore, adding IBIs to the model has little effect in unstructured, well-mixed populations.

### Group-structured populations

**Fig 6:**
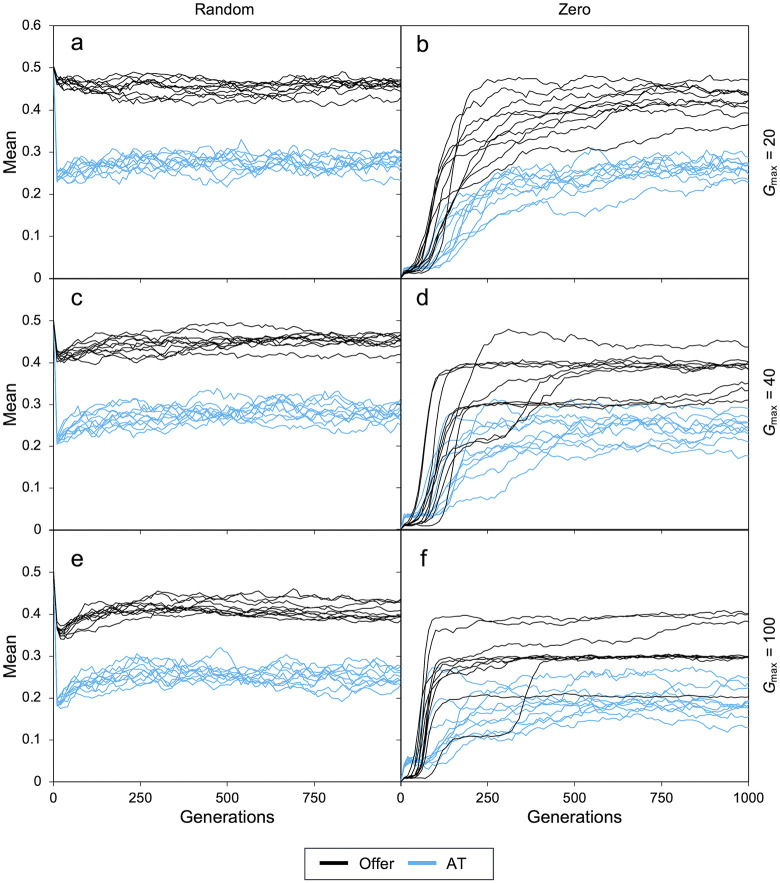
Offer and AT evolutionary trajectories for individual simulations for two initial conditions: Random and Zero in group-structured populations for *G*_max_ = 20 (a, b), *G*_max_ = 40 (c, d), and *G*_max_ = 100 (e, f).

When group structure is introduced (Study 2, Table 3), IBIs have a profound effect on the evolution of mean offers, ATs, and offer-AT gaps compared to well-mixed populations (Fig 6). In group-structured populations, both mean offers and ATs evolved to much higher levels when reproduction was constrained by IBIs. For the Random initial condition, mean offers evolved to over 0.4 with IBIs (Fig 6a, 6c, and 6e). For the Zero initial condition, some simulations evolved over 0.4, but especially for *G*_max_ = 100, several simulations appear to have stabilized around 0.3 and one at 0.2 (Fig 6f). However, even starting from the Zero initial conditions, some simulations consistently achieved the same levels as in the Random initial condition.

**Fig 7:**
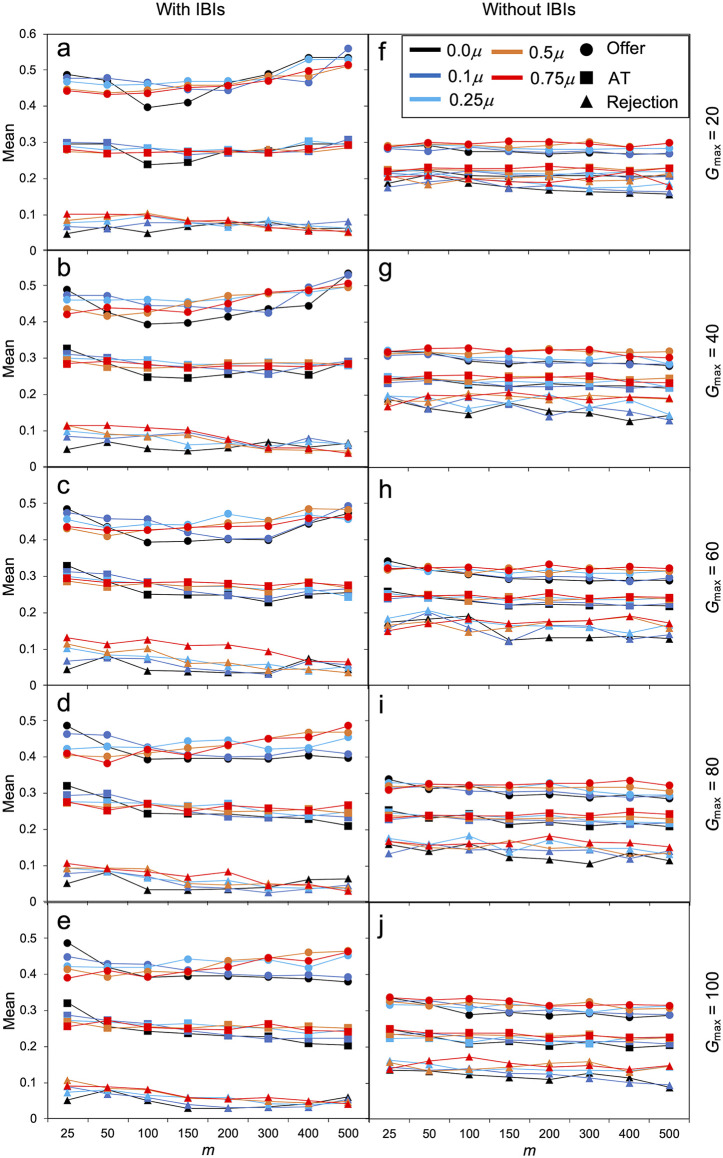
The effect of maximum group size, resource variation, and *m* on measures of UG evolved behavior with and without IBIs. IBI length ranged from *m* = 25 to 500 with **(a-e)** and without IBIs **(f-j)**. The resource standard deviation was varied, *σ*_*µ*_ = 0.0 to 0.75*µ*.

More generally, for the Random initial condition and varying *G*_max_, *m,* and *σ*_*µ*_ for agents with and without IBIs, generous mean offers and wide offer-AT gaps robustly evolved (Fig 7). For agents with IBIs, mean offers evolved close to or above 0.4 with wide offer-AT gaps (Fig 7a-7e). For agents without IBIs, mean offers evolved to approximately 0.3 with much narrower offer-AT gaps (Fig 7f-7j).

**Fig 8:**
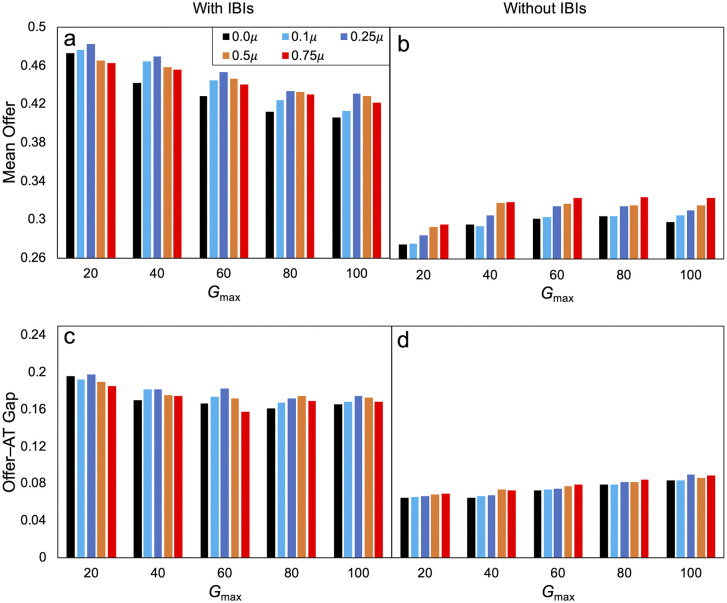
Mean offers and offer-AT gaps averaged over *m.* Data are from Fig 7 for different levels of resource variation (*σ*_*µ*_ = 0.0 to 0.75*µ*) for agents with **(a, c)** and without **(b, d)** IBIs.

Mean offers and offer-AT gaps were averaged over *m* and plotted for different values of *G*_max,_ illustrating the large effect of reproduction constrained by IBIs (Fig 8). No resource variation (*σ*_*µ*_ = 0.0) resulted in overall slightly lower mean offers, while moderate variation (*σ*_*µ*_ = 0.25) produced the highest mean offers, and as variation continued to increase, mean offers dropped slightly for agents with IBIs (Fig 8a). For agents without IBIs, no variation also resulted in the lowest offers, but mean offers tended to increase as resource variation increased (Fig 8b). A similar pattern was observed for offer-AT gaps (Fig 8c-8d). Mean offer-AT gaps ranged from 0.16 to almost 0.2 for agents with IBIs, while they ranged from 0.06 to nearly 0.09 for agents without IBIs.

**Fig 9:**
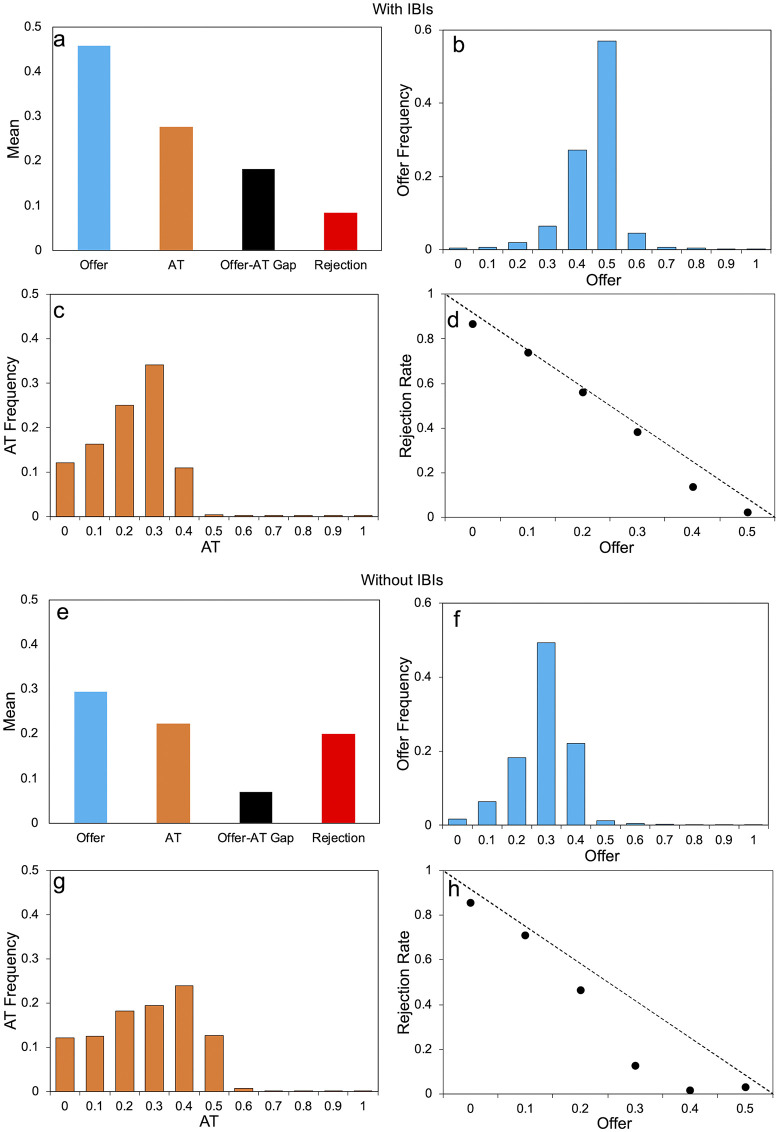
Simulation results for group-structured populations with and without IBIs (*G*_max_ = 20, *m* = 150, and *σ*_*µ*_ = 0.5*µ*). Simulation results with IBIs are plotted for mean offers, ATs, offer-AT gaps, and rejections (a), offer distribution (b), AT distribution (c), and relative rejection rates (dashed lines indicate a linear decline from 1.0 for offers of 0.0 to 0.0 for offers ≥ 0.5, d). Simulation results without IBIs are plotted for mean offers, ATs, offer-AT gaps, and rejections (e), offer distribution (f), AT distribution (g), and relative rejection rates (h).

An example set of parameters with and without IBIs plotted in the same format as Fig 1 is illustrated in Fig 9. With IBIs (Fig 9a), the mean offer, AT, and offer-AT gap are slightly higher than those in Fig 1a, while the mean frequency of rejections is slightly lower than in Fig 1a. Offer and AT distributions in Fig 9b-9c are similar in form to those in Fig 1b-1c as are relative rejection rates (cf. Figs 9d and 1d). For comparison, Fig 9e-9h are corresponding simulations for agents without IBIs. On the one hand, the similarities between Figs 1a-1d and 9a–9d-9d, and on the other hand the dissimilarities between Figs 1a-1d and 9e–9h, strongly suggest that, in the context of group structure, IBIs may be important for explaining the evolution of behavior observed in the UG.

### Mutation rates

**Fig 10:**
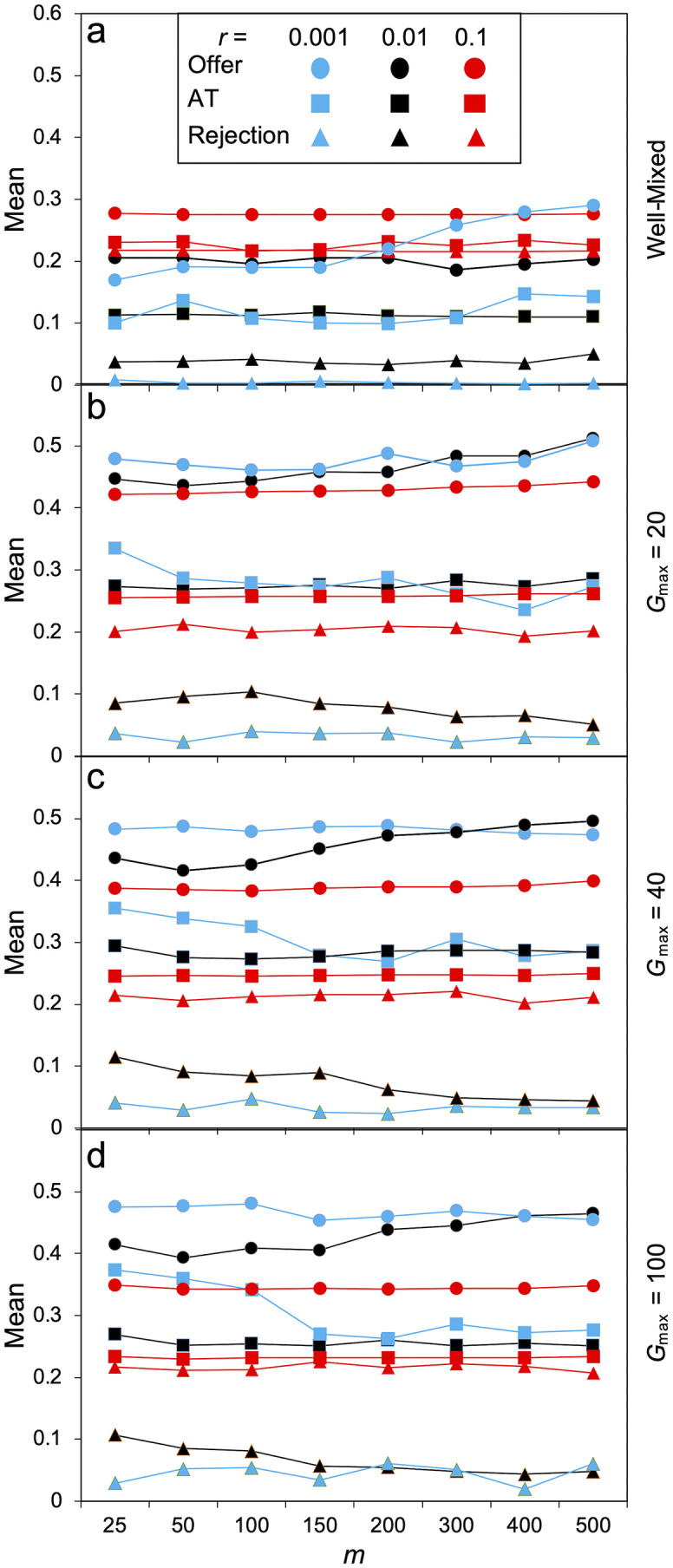
Different mutation rates, *r*, in well-mixed and group-structured populations with IBIs: well-mixed populations (a); group-structured populations, *G*_max_ = 20 (b), *G*_max_ = 40 (c), and*G*_max_ = 100 (d).

We next investigated the effects of different mutation rates in unstructured and group-structured populations (Study 3, Table 3). In unstructured populations, a high mutation rate, *r* = 0.1, increased mean offers by approximately 0.07 compared to *r* = 0.01 (Fig 10a). For the lowest mutation rate, *r* = 0.001, evolved mean offers were similar to *r* = 0.01 for *m* ≤ 200 but close to 0.3 for *m ≥* 300 (Fig 10a).

High mutation decreased mean offers for group-structured populations, especially as *G*_max_ increased (Fig 10b-10d). High mutation also decreased the offer-AT gap as *G*_max_ increased. Mean ATs were slightly higher than 0.2 for all values of *G*_max_. For *r* = 0.001, mean offers were slightly higher for values of *m* ≤ 200, offer-AT gaps were narrower for *G*_max_ = 40, 100, and *m* ≤ 100, and mean rejection rates were generally lower than for *r* = 0.01, especially for *G*_max_ = 20.

**Fig 11:**
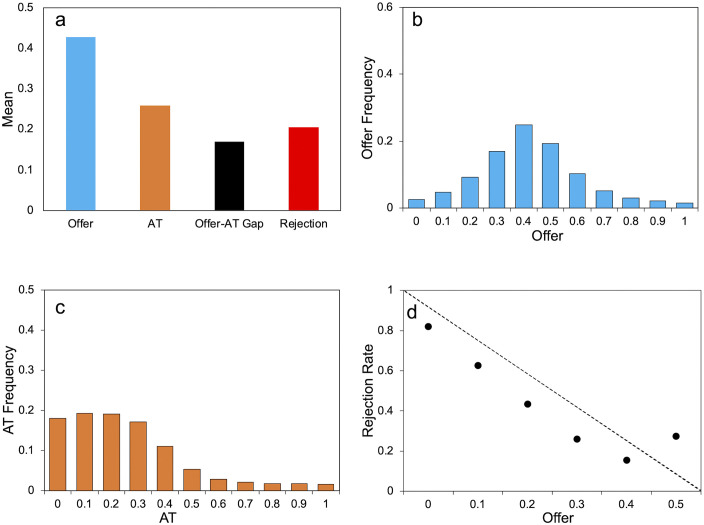
Simulation results for group-structured populations with IBIs and a mutation rate of *r* = 0.1 (*G*_max_ = 20, *m* = 150, and *σ*_*µ*_ = 0.5*µ*). Simulation results are plotted for mean offers, ATs, offer-AT gaps, rejections **(a)**, offer distribution **(b)**, acceptance threshold distribution **(c)**, and relative rejection rates (dashed lines indicate a linear decline from 1.0 for offers of 0.0 to 0.0 for offers ≥ 0.5, **d)**.

Using the same parameter conditions as Fig 9a-9d but increasing from *r* = 0.01 to *r* = 0.1 produced the results depicted in Fig 11. While the evolved mean offer and AT, as well as offer-AT gap, are similar to empirical results and with a higher mean frequency of rejections (Fig 1a), distributions of offers, ATs, and relative rejection rates are not similar in pattern (cf. Figs 11 and 1). Interestingly, if one only compared Figs 11a to 1a, one might conclude that higher mutation rates are at least consistent with some patterns in the empirical data; however, the distributions in Fig 11b-11c are dissimilar to those in Fig 1b-1c, especially in producing right-skewed offer (Fig 11b) and AT (Fig 11c) distributions not observed in the corresponding empirical distributions (Fig 1b-1c). In addition, relative rejections are nonlinear (Fig 11d), where the relative rejection rate for offers of 0.5 or greater were approximately 4% in empirical data (Fig 1d), 2.5% for simulation data with a mutation rate of *r* = 0.01 (Fig 9d), and over 27% for simulation data with a mutation rate of *r* = 0.1 (Fig 11d).

### Dispersion and density

**Fig 12:**
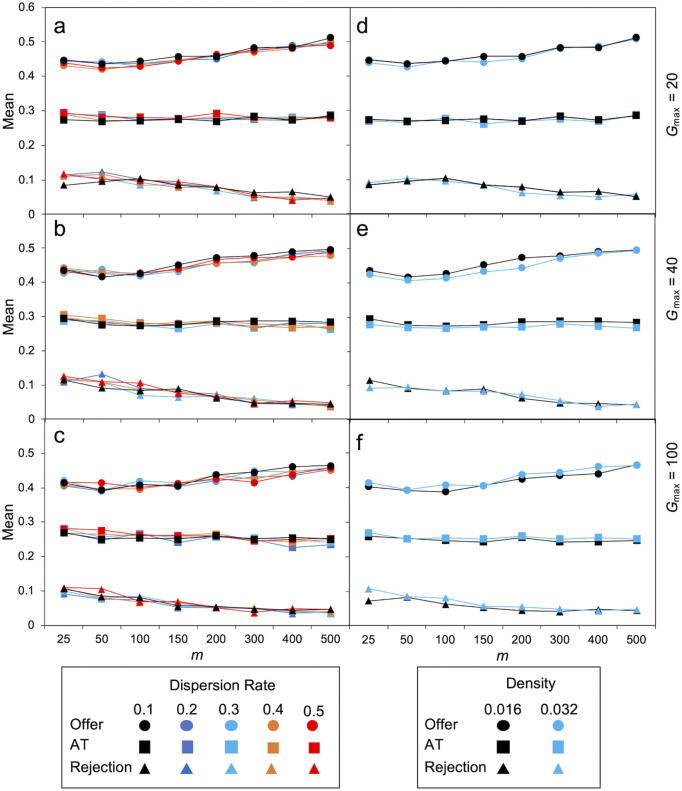
Dispersion and the spatial density of groups had little effect on measures of the UG. Results for mean offers, ATs, and rejections for five rates of dispersion **(a, b, c)**. Results for mean offers, ATs, and rejections for levels of density **(d, e, f)**.

We also investigated whether the dispersion of agents to other groups and the density of groups mattered (Studies 4 and 5, Table 3). Dispersion rates are of particular interest because dispersion mixes agents among groups, reducing relatedness, and thereby may reduce evolved mean offers. However, neither dispersion rates nor increased density substantially affected measures of the UG (Fig 12). That dispersion rates as high as 0.5 did not matter was surprising, since in well-mixed populations, mean offers and ATs evolved much closer to the Nash equilibria. This suggests that the evolution of generous offers could have occurred under a broad range of dispersal rates in hunter-gatherer populations.

### Asexual reproduction

**Fig 13:**
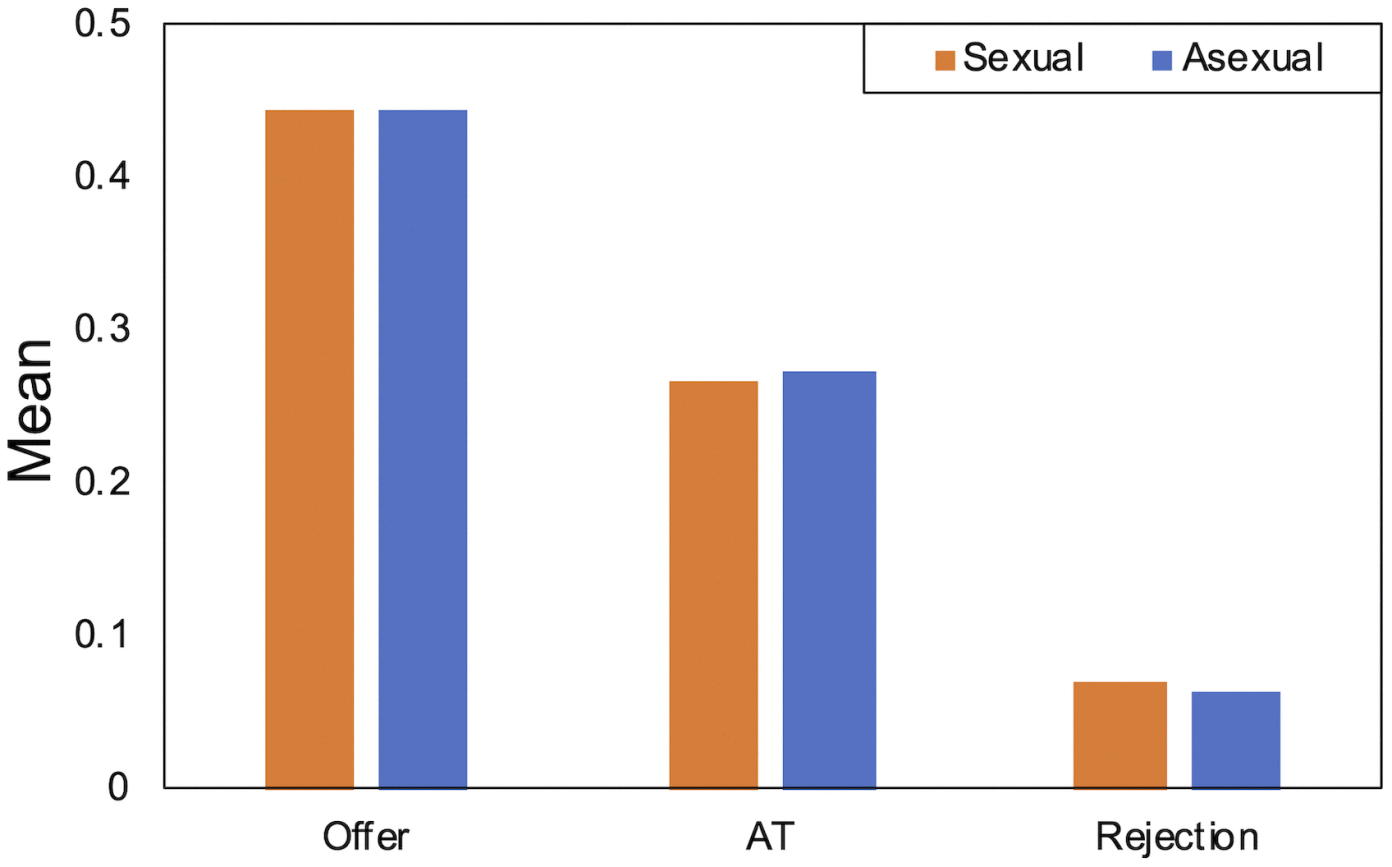
Comparison of asexual and sexual reproduction group-structured populations averaged over *G*_max_ = 20 to 100, *m* = 25 to 500, and *σ*_*µ*_ = 0.0 to 0.75*µ.*

We reasoned above in developing the model that sexual reproduction may yield different results than asexual reproduction and that sexual reproduction should be superior in this context (Study 6, Table 3). However, and somewhat surprisingly, we found no differences when we ran the same group-structured simulations with asexual reproduction (Fig 13), demonstrating that these results are not sensitive to the mode of reproduction.

### Rejections and population size

**Fig 14:**
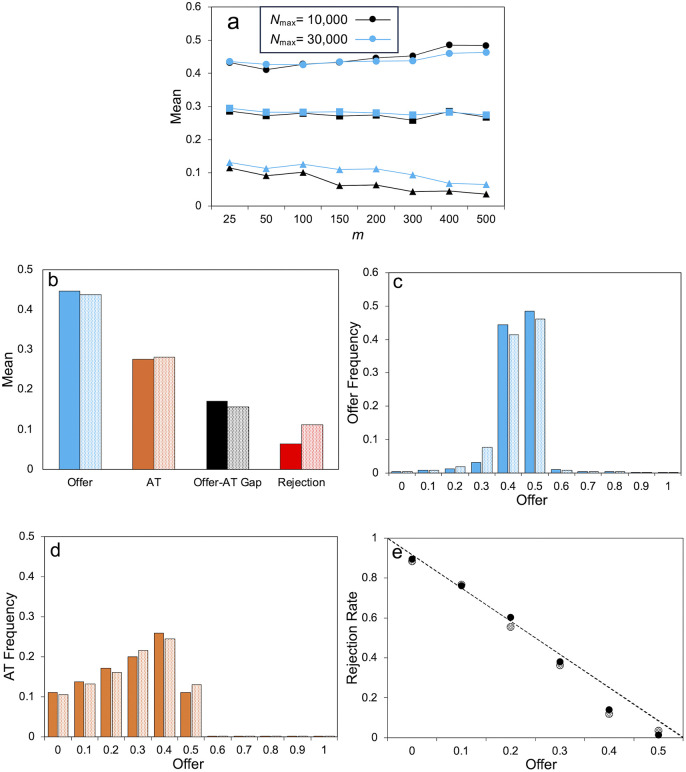
Comparison of simulations with different population sizes (*G*_max_ = 60, *m* = 200, and *σ*_*µ*_ = 0.5*µ*). Simulation results are plotted for mean offers, ATs, offer-AT gaps, and rejections **(a)**, offer distribution **(b)**, AT distribution **(c)**, and relative rejection rates (dashed lines indicate a linear decline from 1.0 for offers of 0.0 to 0.0 for offers ≥ 0.5, **d)**. Results for *N*_max_ = 10,000 are solid colors, and for *N*_max_ = 30,000 are patterned colors **(b-d)**.

Mean rejections for simulations with IBIs were, in general, lower than the empirical values depicted in Fig 1a. Indeed, mean rejection decreased as *G*_max_ increased and as *m* increased. The number of groups in a population is a function of population size. That is, as *G*_max_ increases while holding *N*_max_ constant, the number of groups in a population decreases. We reasoned that increasing *N*_max_ relative to *G*_max_ would lead to an increase in mean rejections, as more groups would allow more inter-group variation in offers and ATs. To test this conjecture (Study 7, Table 3), we ran a series of simulations with *G*_max_ = 60 and three times the population size, *N*_max_ = 30,000, which yielded a similar number of groups as those with *G*_max_ = 20 and *N*_max_ = 10,000. Tripling the population size had little effect on UG measures, except for mean rejections, which increased by 43% with a larger population size (Fig 14). In contrast, mean offers decreased by 2% and ATs increased by 2% with larger population size.

### Resource shortages and surpluses

We assessed how resource shortages and surpluses relative to reproductive thresholds, *θ*, affect the evolution of mean offers and ATs for agents with IBIs (Study 8, Table 3). We introduce a coefficient *α* such that for *α* > 1.0 (*αθ* > *θ*), there are resource shortages, and for *α* < 1.0 (*αθ* < *θ*), there are resource surpluses for agents, both relative to IBI length *m*.

**Fig 15:**
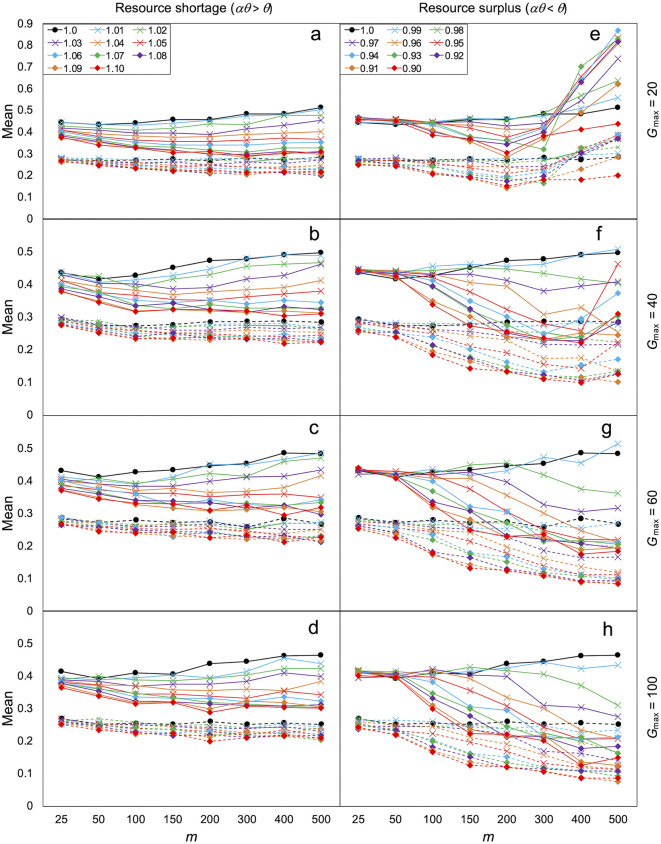
The effects of resource shortages and surpluses, group size, and *m* on evolved offers (solid lines) and ATs (dashed lines) with IBIs. Resource shortage ranged from *α* = 1.01 to 1.1 in increments of 0.01 **(a-d)** and resource surplus *α* = 0.99 to 0.9 in increments of 0.01 **(e-h)**. The resource standard deviation was varied, *σ*_*µ*_ = 0.5*µ*. The vertical axis is 0.0 to 0.9 for a and e and 0.0 to 0.6 for the remainder.

Resource shortages reduced both evolved mean offers and ATs (Fig 15a-15d). For *m* ≥ 200 and large resource shortages (*α* = 1.08, 1.09, and 1.1), populations evolved mean offers and ATs close to populations without IBIs (cf. Figs 15a-15d, 7f–7h and 7j). However, for *m* < 200, evolved mean offers and ATs did not drop as low, especially low values of *m* (*m* = 25 and 50; cf. Figs 15a-15d, 7f–7h and 7j). For small resource shortages (*α* = 1.01 and 1.02), evolved mean offers were close to, or slightly below, *α* = 1.0. Evolved mean ATs exhibited a similar pattern (Fig 15a-15d).

Resource surplus resulted in complex nonlinear interactions between *m* and *G*_max_. First, (i) for *G*_max_ = 20 and low values of *m* (*m* = 25 and 50; Fig 15e), mean offers evolved close to the same level as *α* = 1.0; (ii) for large values of *m* (*m* ≥ 400 and 1.0 > *α* > 0.9), mean offers evolved above 0.5, reaching 0.87 (for *α* = 0.94, *m* = 500); (iii) but for *α* = 0.9 and *m* = 500, mean offers evolved below *α* = 1.0; (iv) for intermediate values of *m* (50 < *m* < 400 and 0.97 > *α* > 0.9), mean offers evolved below *α* = 1.0; and (v) for *α* = 0.99, evolved mean offers were close to *α* = 1.0 (Fig 15e). The evolution of mean ATs followed a similar pattern (Fig 15e). Second, for *G*_max_ > 20 and low values of *m* (*m* = 25 and 50; Fig 15f-15h), mean offers evolved to levels close to *α* = 1.0, but unlike *G*_max_ = 20 and *m* > 50 (with some exceptions at *G*_max_ = 40), mean offers evolved to much lower levels than for *α* = 1.0. Indeed, especially for *G*_max_ = 100 and large values of *m* (*m* ≥ 300 and *α* ≤ 0.96), mean offers often evolved to levels observed in well-mixed populations under Random initial conditions, and even to similar levels for Zero initial conditions (Fig 4). For a 1% resource surplus (*α* = 0.99), evolved mean offers were close to those for *α* = 1.0. The evolution of mean ATs followed a similar pattern (Fig 15f-15h).

### Odd groups

During a simulation, the number of agents in a group changes from even to odd, or vice versa, when agents die, are born, or disperse in and out of groups (Study 9, Table 3). We assumed that each agent plays a UG with another randomly selected agent in its group only once per timestep. This creates a problem for odd groups. What happens to the odd agent who cannot pair with another agent that has not already played on a given timestep? Selection on strategies should not depend on whether the number of agents in a group is even or odd. Therefore, any selection differences between being in a group with an even number of agents versus an odd number of agents should be minimal. We considered four plausible rules for handling the odd agent in a group. The rule that best minimizes selection differences between odd and even groups is the half-game rule (HGR): the odd agent is either a proposer or a responder 50% of the time and plays another randomly selected member of its group who has already played. It keeps its payoff, but the other agent does not. Thus, all play a UG on each step, and because only the odd agent keeps its payoff, all agents in an odd group have the same expected payoff as agents in even groups. Alternatively, the odd agent could play under the full-game rule (FGR), and both agents keep their payoffs. This would allow all agents to play at least one UG, but one agent would play two UGs on a given time step, increasing the expected payoff for all agents in odd groups relative to even groups. This would create a resource surplus, as in the previous section, which should alter the outcome of evolutionary simulations. A third rule again assigns the role of proposer or recipient to the odd agent 50% of the time, but as a proposer, it keeps the endowment, and as a recipient, it receives nothing. This keep rule (KR) would maintain the same expected payoffs for agents in odd and even groups (if all agents make the same offer and all offers are accepted), but would alter the selection conditions between these groups because an odd agent does not play a UG. Finally, there is the no-play rule (NPR), where the odd agent does not play and receives nothing. This rule would violate the assumption that all agents play a UG at every time step, and the expected payoff for an agent in an odd group would be lower than that in even groups.

**Fig 16:**
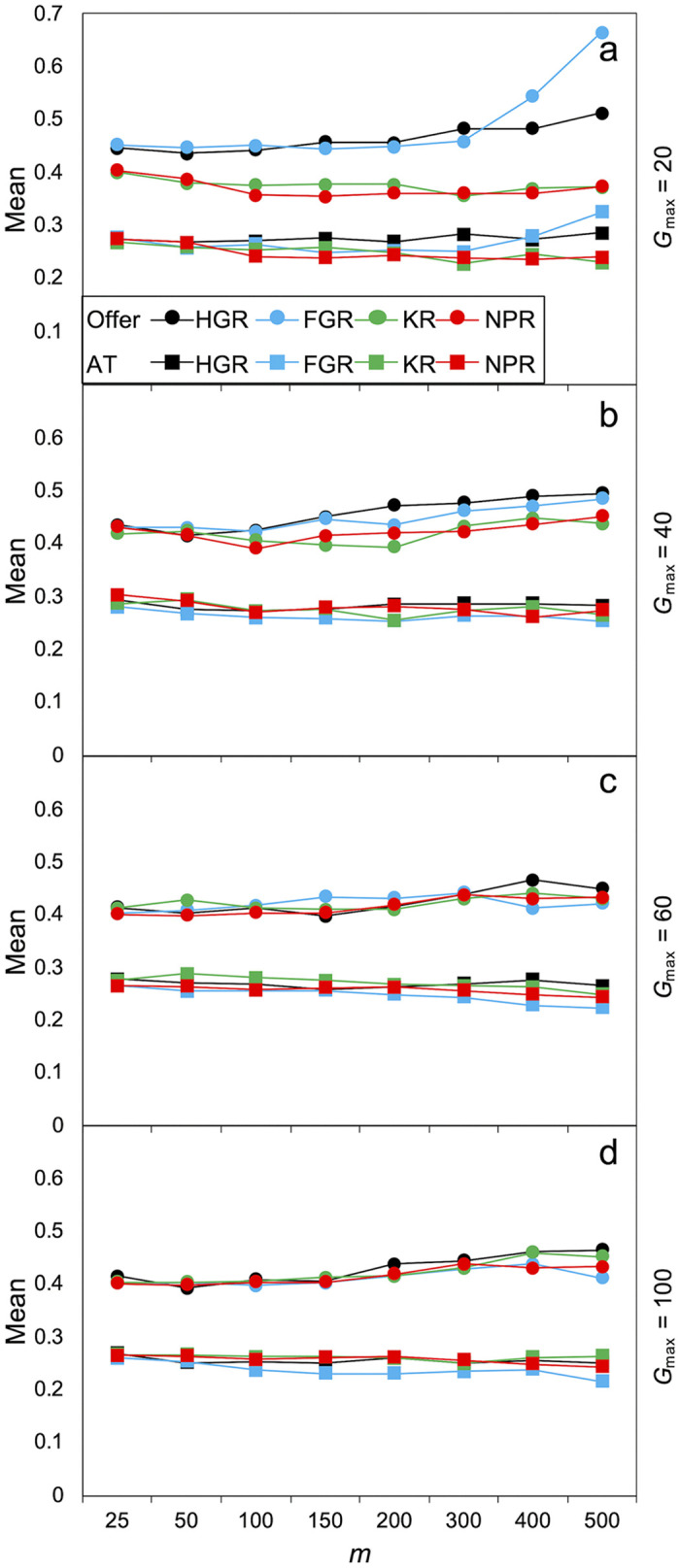
The effects of different rules for groups with odd numbers of agents in relation to *m* and *G*_max_. The four rules depicted are HGR **(a)** used in all simulations above, FGR **(b)**, KR **(c)**, and NPR **(d)**. The vertical axis in all figures ranges from 0.0 to 0.7.

The effects of these different rules on evolutionary outcomes should be greatest for small groups and diminish as group size increases because the change in expected payoffs and the likelihood of being the odd agent decreases as *G*_max_ increases. To investigate these effects, we systematically ran simulations using these four rules across different group sizes, as illustrated in Fig 16. These rules have substantial effects on the evolution of mean offers and ATs for the smallest group size (*G*_max_ = 20), but for larger group sizes (*G*_max_ > 20), evolved mean offers and ATs tend to converge.

## Discussion

### IBIs and group structure

Simulations of group-structured populations with reproduction limited by IBIs aligned with patterns observed in the experimental data. In contrast, simulations of well-mixed populations with or without IBIs more closely aligned with patterns predicted for  Nash equilibria, illustrating the importance of group structure in accounting for patterns observed in the experimental data. In group-structured populations without IBIs, although mean offers evolved to higher levels than in well-mixed populations, they aligned much less well with the empirical data than simulations with IBIs. These results underscore the synergistic evolutionary effect of combining group structure and IBIs.

### Offer costs, rejection costs, and relative endowment size

Two types of costs occur when playing the UG: offer costs and rejection costs. For proposers, offer costs are the resources they give to responders. Offer costs are reduced by making smaller offers. Rejection costs occur when both parties end up with nothing when offers are rejected. Rejection costs penalize both players but are more costly to proposers when offers are less than 50% of a resource. Rejection costs create selection pressure for offers and ATs that minimize rejections. The Nash equilibrium minimizes both offer and rejection costs, and we observed that both costs tend to be minimized in well-mixed populations (see Figs 4 and 5) and under certain conditions with resource surpluses (Fig 15g and 15h).

The resource endowment size relative to the resources required to reproduce, *θ*, decreases as *m* increases. For example, if *m* = 25, the mean endowment was 4% of the resources required to reproduce, while for *m* = 500, it was 0.2%. The former is 20 times the latter, implying that the two types of costs in the UG decrease as *m* increases.

When group structure is introduced, it subdivides a population and restricts the number of agents with which an agent can interact. We assumed that agents accumulate *θ*, Eq (2), resources during an IBI with *m* games, provided that all offers are accepted and agents use the same offer strategy, thereby allowing for the maximum expected reproduction rate of individuals in a group. Groups that minimize rejection costs produce more offspring, fission more often, and survive longer. This is because rejection costs are an absolute loss of resources, and the number of offspring produced in a group is proportional to the available resources. The more rejections in a group, the fewer offspring are produced due to an absolute loss of resources. For example, in a group with 10 members, if agents play *m* UGs to accumulate the expected resources during an IBI, but one member has an AT greater than all the others’ offers, at least 9 of the 10 members would be expected to receive 10% or less of the resources they would accumulate over *m* games if there were no rejections. Members of such groups would have substantially lower reproductive output than those in a group with fewer systematic rejections. In the context of groups, wide offer-AT gaps provide a kind of insurance against rejection costs when rejection costs are relatively higher than offer costs.

Group structure can therefore increase the importance of rejection costs, and groups with members who have wide offer-AT gaps tend to avoid rejection costs as a function of the gap size. In simulations where agent reproduction is unconstrained by IBIs, group structure facilitates higher mean offers and broader offer-AT gaps than in well-mixed populations (Fig 7f-7j), but not nearly as high or as large as when agent reproduction is constrained by IBIs (Fig 7a-7e). Because IBIs reduce the benefit of making stingy offers, rejection costs become more important, resulting in higher mean offers and broader offer-AT gaps. In short, IBIs limit offer costs, allowing rejection costs to push mean offers higher and increase the offer-AT gap to reduce rejections. This also appears to explain why the relatively high dispersion rates have little effect on the evolution of mean offers, offer-AT gaps, and other measures in this model.

Returning to the synergistic effect of combining group structure with IBIs on the evolution of generous offers and wide offer-AT gaps, they can be explained by (i) being in a group and (ii) the relative reduction in offer costs compared to rejection costs that comes with IBIs. Agents in groups with fewer rejections produce more offspring than those with more rejections because offspring production is proportional to available resources. IBIs reduce offer costs by limiting reproduction rates, thereby reducing the benefit of making low offers to exploit wide offer-AT gaps. With decreased offer costs, rejection costs become more salient and are reduced by increasing the offer-AT gap with more generous offers. Consequently, groups with more generous offers and wider offer-AT gaps produce more offspring than groups with less generous offers and narrower offer-AT gaps. Thus, wider offer-AT gaps provide insurance against rejections and thereby increase the reproductive rate of agents in such groups.

Long-term and systematic resource shortages or surpluses influence the evolution of mean offers, ATs, and offer-AT gaps in complex ways. For resource shortages (1.0 < *α* ≤ 1.1), if all offers are accepted and agents have the same offer strategy, there are fewer than expected resources to achieve the resource threshold, *αθ*, in *m* UG games. This creates opportunities for less-generous strategies to accumulate resources more rapidly by making stingier offers, thereby lowering evolved mean offers and reducing the offer-AT gap (Fig 15a-15d). For resource surpluses (1.0 > *α* ≥ 0.9), if all offers are accepted and agents have the same offer strategy, there are more resources than required to achieve the resource threshold, *αθ*, in *m* UG games. This allows some rejections (depending on *α*) without reducing the expectation of achieving *αθ* in *m* UG games, which reduces rejection costs for making stingy offers. This could explain the low evolved mean offers observed for *G*_max_ > 20, *m* > 100, and *α* ≤ 0.95 (Fig 7f-7j) when coupled with the relative decrease in costs with increasing *m*; however, there are two striking exceptions to this reasoning.

First, for *G*_max_ = 20, mean offers (*α* ≤ 0.97 and 50 < *m* ≤ 300) are lower than when *α* = 1.0, similar to the pattern observed for *G*_max_ > 20. However, for large *m* (*m* = 400, 500, and 1.0 > *α* > 0.9), mean offers evolved to extraordinarily high levels. Even though mean ATs also evolved higher, the offer-AT gap is greater than for *α* = 1.0, indicating that avoiding rejections was an important factor in evolving high mean offers. The costs are the same as when *G*_max_ > 20, with a relative decrease in costs as *m* increases. Here, group size may play a crucial role. For *G*_max_ = 20, there were about 9 agents on average per group (see Fig A in S2 Text). One possible explanation is that, with an average of 9 agents per group, it is more likely that at least one “bad” agent will have a relatively high AT, resulting in a large number of rejections and thereby significantly reducing the reproductive output of that group. With greatly reduced costs for *m* ≥ 400, the increased likelihood of a “bad” agent drives up mean offers and increases the offer-AT gap, while for *G*_max_ > 20, the lower likelihood of a “bad” agent allows lower offers to evolve as we argued above.

Second, for all *G*_max_, 1.0 > *α* ≥ 0.9, and *m* = 25 and 50, similar mean offers evolved, though proportionally lower ATs evolved as *α* decreased. This may be due to the relatively small number of games, *m*, played during an IBI, resulting in relatively higher resource endowments per UG. This would result in relatively higher costs per UG and in greater inter-agent variability in resource accumulation due to chance differences in the roles played (i.e., proposer or responder), as previously noted in the context of including IBIs in the evolution of offers in the dictator game [67]. Higher costs with higher relative endowments per UG may also explain why evolved mean offers did not decrease as much for *m* = 25, 50 as for *m* ≥ 100 for resource shortages (Fig 15a-15d). The importance of the relative size of the resources bargained for in the UG is also illustrated in Fig 16. Alternative rules FGR, KR, and NPR all converge on the same results as HGR for *m* = 25 and 50.

An important caveat regarding resource shortages and surpluses in this analysis is that, in these simulations, both were assumed to persist over an evolutionary time frame. Likely, the evolutionary time-frame characteristics of the environment would alter the optimal length of IBIs to match expected resource availability. Resource surpluses resulting from adaptations such as bipedality, prosocial behavior, larger brain size, and the evolution of language should reduce the length of IBIs [72,81]. Counterfactually, resource shortages would suggest that some of the previous adaptations failed to evolve. Theoretically, therefore, the length of IBIs should evolve to optimize the use of available resources. Nevertheless, resource shortages or surpluses on shorter time scales could facilitate more rapid and local changes in offers, ATs, and offer-AT gaps if more rapid adaptive processes are operating (e.g., cultural evolution or social learning, see below for further discussion).

Nevertheless, in all resource surplus conditions, for *m* = 25 or 50, evolved mean offers were essentially the same for all surplus conditions and *G*_max_. This implies that when relative resource endowments are high, generous offers and wide offer-AT gaps will consistently evolve across different group sizes, even with resource surpluses. When there are resource shortages, the decrease in offer generosity and the offer-AT gap are smaller for high relative resource endowments. This suggests that, in the face of changing resources and group sizes, if relative resources are high in the UG, generous offers and wide offer-AT gaps would robustly evolve.

### Variation in UG results across studies and societies

Differences in mean offers, mean ATs, offer-AT gaps, mean rejections, and offer and AT distributions are modulated by parameters *G*_max_, *m,* and *σ*_*µ*_ (Fig 7). Parameters such as these and others vary across studies and societies. Forty-four UG experiments reported by Cochard et al. [7] had mean offers in the range of 0.45 to 0.5, and one study had a mean of 0.57 (Fig B in S2 Text). Indeed, almost 92% of these experimental studies fell in the range 0.34 to 0.57 (Fig B in S2 Text), and all of the offer means in Fig 7 fell in the range of 0.34 to 0.56. Acceptance thresholds and offer-AT gaps vary greatly across societies (Fig C in S2 Text). Acceptance thresholds and offer-AT gaps also varied in these simulations (Fig 7), though to a lesser extent than in Fig C in S2 Text. Thus, another strength of this modeling approach is that it can potentially account for some of the variation in UG measures across studies and societies.

### Mutation rates

When the mutation rate is increased in populations with IBIs, the simulation results did not align as well with patterns in the experimental data. Unlike lower mutation rates, for *r* = 0.1, mean offers decreased as *G*_max_ increased. This suggests that high mutation rates or noise cannot adequately explain human behavior in the UG in the modeling approach presented here (see Figs 10 and 11). Akdeniz and van Veelen [10] identified complementary issues related to high mutation rates in their critical review of mutation-selection models.

For instance, in the Rand et al. [25] evolutionary model of the UG, each agent plays against every other agent in the population in a round-robin format at each time step. The average payoff for each agent, *π*_*i*_, was calculated at each time step, and selection was proportional to exp(*wπ*_*i*_), also known as a softmax distribution, where *w* represents the selection intensity. Successful offspring inherit their parents’ strategy (*p* and *q*), where *p* and *q* are randomly mutated with probability *r*. The resource bargained for in each UG was *x* = 1, but the average payoff, *π*_*i*_, per agent was less than 1. In Fig D in S2 Text, we have plotted a hypothetical average payoff distribution for 100 agents where payoffs were randomly drawn from a uniform distribution with the range [0, 0.5], assuming the maximum value of *π*_*i*_ is 0.5. For selection intensities of *w* = 0.001, 0.01, and 0.1, exponential normalization greatly reduces variation in probabilities among agents (Fig D in S2 Text). For *w* = 1.0, we see some variation among agents in probabilities, but it is not till *w* = 5.0 that we see a normalized exponential probability distribution with somewhat similar variation as in the linear normalized probability distribution in the top left of Fig D in S2 Text. Selection is exponentially weak for selection intensities *w* < 1, resulting in approximately uniform probability distributions. This implies that any offer strategy will have nearly the same chance of being chosen for the next generation. For exponentially weak selection, mutation dominates the evolutionary process, resulting in a uniform or nearly uniform distribution of offers with a mean close to 0.5.

Akdeniz and van Veelen [10] extensively analyzed the consequences of this kind of selection model and its interaction with mutation. As expected from Fig D in S2 Text, the exponential normalization of payoffs for agents results in random walks of mean offers and ATs with uniform distributions of offers and ATs (see Fig 2; Akdeniz and van Veelen [10]). As selection intensity increases (i.e., *w* > 1), selection overcomes mutation, and mean offers and ATs evolve towards the Nash equilibrium, as expected. Akdeniz and van Veelen’s [10] critical analysis implies that we must develop more realistic selection models to make progress in explaining human behavior in the UG. The more biologically realistic selection mechanism in the current model behaves very differently at varying mutation rates and therefore cannot be adequately represented by the less realistic and more simplified mutation-selection models using normalized exponential probability distributions.

The importance of biological and social realism has implications for the mechanisms and constraints that may or may not be essential to include in future models and experimental studies. For example, Hintze and Hertwig [64] extended the Rand et al. [25] mutation-selection model to include the evolution of kin recognition. Introducing the evolution of kin recognition enabled the evolution of generous offers without extremely weak selection. However, kin relationships may be relatively unimportant in the biologically more realistic model presented here. Generous offers with wide offer-AT gaps evolved even under high dispersion and for large numbers of agents in groups with sexual or asexual reproduction. Because there is more mixing of agents with higher dispersion and larger groups, the evolution of generous offers and wide offer-AT gaps is driven by the interaction of group structure and IBIs and, to a lesser extent, if at all, by relatedness.

### Realism

There are serious challenges to developing more realistic selection models. Perhaps the most obvious is the complexity introduced by modeling biologically more realistic mechanisms. For computational models such as this one, increased realism implies not only increasing the number of parameters but also the computational resources needed to simulate models with increased realism. Increased realism can lead to intractable models and the increased likelihood of introducing systematic errors and biases. A deeper issue is how to decide which of the indefinitely many mechanisms, processes, and behaviors to include in a model. How much realism is needed to adequately model the evolution of human behavior? If this model is on the right track toward greater realism, what alternative paths might yield additional theoretical insights? All models are false or simplifications for many dimensions of realism [68,82]. Realism in modeling is relative and comes in degrees. Introducing IBIs is a relative increase in realism, but no physiological details of IBIs were included, so the introduction of IBIs is a relative increase in realism, but with extreme simplification. The same can be said of other aspects of this model, such as group structure, inheritance, and reproduction, as well as interpersonal bargaining modeled by the UG. Paths to increased explanatory realism, either starting from this model or through alternative paths, are needed to achieve truer theories [68].

### Future directions

Some UG behavior is heritable [77] and may be mediated by evolved psychological or social mechanisms, such as reputation, spite, or empathy, that explain generous offers and other UG behaviors. Such mechanisms raise the evolutionary question of how they could have evolved. If population structure and IBIs synergistically interact as in this model, then psychological and social mechanisms favoring generous offers and wide offer-AT gaps, (e.g., empathy, a sense of or preference for fairness, or aversion to unfairness or inequity, or reputation) could have evolved to facilitate optimal behavior especially in the dynamic biological and social conditions (e.g., changes in group structure or frequency of bargaining events) humans evolved. Building on this logic, a logical next step would be to use this modeling approach to explore whether social and psychological mechanisms could evolve to help agents adopt or learn optimal strategies for adapting to changing biological and social environments. For instance, this could involve modeling the evolution of social play learning mechanisms that provide information about future adult cooperative contexts [83]. In short, although we did not make assumptions about psychological mechanisms to explain the evolution of generous offers and wide offer-AT gaps in the UG, this model suggests that they could have been shaped by natural selection during human evolution.

Cultural evolution (e.g., Bowles and Gintis [84]) could also explain at least some behavior in the UG. The central result of modeling IBIs and group structure was that agents evolved behavior robustly similar to empirical data from UG studies. This implies that generous offers and wide offer-AT gaps are advantageous under these conditions, and that cultural evolution, via social learning, could be a process for rapidly evolving norms for making offers and deciding what to accept. The simulations on resource shortages and surpluses demonstrated that the costs of offers and ATs can dramatically change when there are resource shortages or surpluses (see Fig 15). Cultural evolution, as a more rapid process of behavioral change, could facilitate more rapid and adaptive changes in UG bargaining behavior in response to ecological-time-scale shifts in resource availability.

However, developing a social learning model that integrate IBIs is not a straightforward adoption of social learning dynamics from the relevant literature. For example, the probability *p*(*i*, *j*) that agent *i* imitates agent *j* is often modeled using a Fermi function in Eq (6).


$$\begin{array}{c} p(i,j)=\frac{\text{1}}{\text{1}+e^{-\beta_{t}(F_{j}-F_{i})}} \end{array}$$ (6)

where *β* is the strength of imitation, and *F* is the fitness of *i* and *j,* where *F* is interpreted as strategy payoffs in a game [85]. If *β* is large, imitating more fit agents is highly probable, but as *β* approaches zero, imitation becomes a coin flip independent of fitness. This suggests that using imitation with low *β* values should replicate the findings of Rand et al. [25], but raise issues similar to those encountered with mutation-selection using softmax distributions. Implementing social learning as imitation in the current model would require more work on possible imitation mechanisms. In the current model, an agent’s fitness is the number of offspring they produce, which depends not merely on the resources or payoffs obtained from playing the UG but also on IBIs and group structure. Thus, imitation models such as (6), based solely on payoffs, would not be compatible with an IBI approach.

This model was inspired by our hunter-gatherer ancestors and their group structure. However, hunter-gatherer societies are organized into multi-level networks that range from small family units to regional populations of more than 800 people [86]. Thus, one dimension for increasing realism would be to develop multi-level network models to investigate whether similar results are observed in the evolution of UG behavior within larger multi-level network structures. Since the time when hunter-gatherer societies dominated human populations, societies have become more multi-level and larger. In short, more realistic population structures, social learning, and the evolution of psychological mechanisms are promising directions for modeling human behavior in the UG.

## Conclusions

Inspired by our hunter-gatherer evolutionary history, we developed a simple model to represent IBIs and group structure. The model demonstrates that, within the context of group structure, IBIs may be crucial for explaining human behavior in the UG and for understanding variation in that behavior across societies. Our aim was to investigate whether more realistic selection models could yield better explanations of data from UG experiments. We found that IBIs modulate offer and rejection costs in the context of group structure, making fitness a complex emergent property of these interactions as well as of group size, resource availability, and the relative size of endowments. In the context of group structure, generous offers and the wide offer-AT gap evolved robustly, insuring against rejection costs. More generally, incorporating IBIs into evolutionary game-theoretical models may prove theoretically fruitful for explaining prosocial behavior. Finally, though challenging, we suggest that a deeper understanding of prosocial behavior, such as fairness and cooperation, requires further theoretical exploration of the dimensions of realism, including biological, social, and psychological factors that can shape human behavior in evolutionary models.

## Supporting information

S1 Text Origin of the data graph. (PDF)

S2 Text Figs A to D. (PDF)
