# Supplementary material for: Evolution and the ultimatum game: An agent-based model with interbirth intervals and population structure
Source: PLoS Comput Biol. 2026 Jun 16;22(6):e1014387. doi: 10.1371/journal.pcbi.1014387 (PMC13289883; doi:10.1371/journal.pcbi.1014387)
Supplement: S1 Text — (PDF) [file pcbi.1014387.s001.pdf]

S1 Text. **Origin of the data graph**

The four graphs in Fig 1 were derived from five sources of UG experiments (Oosterbeek et al. [1]; Cochard et al. [2]; Tisserand et al. [3]; Cooper et al. [4]; Akdeniz and van Veelen [5]). Below, we briefly describe the studies corresponding to the graphs in Fig 1a-d, together with the data plotted.

In Fig 1a, the mean offer of 41.5% is from Cochard et al. [2], which includes UG studies performed in 30 countries. Oosterbeek et al. [1] conducted a meta-analysis of 37 papers containing 75 studies on UG experiments conducted in 25 countries between 1982 and 2003. Studies were excluded if there was no money at stake, proposers had highly restricted offer space, there was one-sided uncertainty, group decision-making, three-person UG, or a lack of anonymity. The mean AT of 27.1% was calculated from 2,629 data points from multiple studies in Akdeniz and van Veelen [5]. The offer-AT gap is the difference between the mean offer (41.5%) and the mean AT (27.1%). The mean rejection rate of 16% is from Oosterbeek et al. [1].

In Fig 1b, the offer distribution is from Tisserand and Cochard [3], who conducted a meta-analysis of 97 studies from 41 papers and a book. This meta-analysis and Cochard et al.'s [2] meta-analysis covered largely the same studies. They obtained individual observations when possible and 2,266 offer data points for the UG. Using the total number of cycles reported and Figure 4 in Tisserand and Cochard [3], we estimated the offer frequency in 10% intervals from 0% to 100%.

In Fig 1c, the AT frequency distribution was calculated from 2,629 data points from multiple studies provided by Akdeniz and van Veelen [5].

In Fig 1d, rejection rates were obtained from Cooper et al. [4], who conducted a meta-analysis of seven papers to investigate the dynamics of responder behavior over repeated rounds

of play. Studies were from standard ultimatum games with one proposer and one responder played anonymously. Studies using the strategy method were excluded, and offers were made in increments of at least 1/10. All studies included at least 10 rounds of play, and proposers and responders maintained their respective roles throughout. There were 387 participants across all of the studies. Their results for the first 10 rounds (3,866 individual observations) were graphed as acceptance rates (Fig 1 in Cooper et al. [4]), which we converted to rejection rates, where the rejection rate for an offer is 1 – the acceptance rate. Two rejection rates are plotted: solid black circles connected with lines represent the rejection rates for responders in rounds 1-5 of play, while solid blue circles indicate the rejection rates for rounds 6-10. Offers are binned into six intervals, with the last interval being greater than or equal to 50%. The rejection rates for rounds 6-10 are significantly higher for the first two categories of offers (i.e., $0 \leq O < 10$ and $10 \leq O < 20$), whereas there is no difference for the remaining offer categories (Cooper et al. [4]). Cooper et al. [4] report a significant change in responder behavior, but the difference is surprisingly small. Cooper et al. [4] acknowledge that the change in responder behavior is minor but conclude that experience probably would not result in convergence to the subgame perfect equilibrium. Instead, responders are slightly more likely to reject low offers.

References

1. Oosterbeek H, Sloof R, Van De Kuilen G. Cultural differences in ultimatum game experiments: evidence from a meta-analysis. Exp Econ. 2004; 7: 171–188. doi:10.1023/B:EXEC.0000026978.14316.74

2. Cochard F, Le Gallo J, Georgantzis N, Tisserand JC. Social preferences across different populations: meta-analyses on the ultimatum game and dictator game. J Behav Exp Econ. 2021; 90: 101613. doi:10.1016/j.socec.2020.101613
3. Tisserand J-C, Cochard F, Le Gallo J. Altruistic or strategic considerations: a meta-analysis on the ultimatum and dictator games. CRESE, Université de Franche-Comté. 2015. Available from: https://api.semanticscholar.org/CorpusID:52576084
4. Cooper DJ, Dutcher EG. The dynamics of responder behavior in ultimatum games: a meta-study. Exp Econ. 2011; 14: 519–546. doi:10.1007/s10683-011-9280-x
5. Akdeniz A, van Veelen M. Evolution and the ultimatum game. Games Econ Behav. 2023; 142: 570-612. doi:10.1016/j.geb.2023.08.005
