# Supplementary material for: Evolution and the ultimatum game: An agent-based model with interbirth intervals and population structure
Source: PLoS Comput Biol. 2026 Jun 16;22(6):e1014387. doi: 10.1371/journal.pcbi.1014387 (PMC13289883; doi:10.1371/journal.pcbi.1014387)
Supplement: S2 Text — (PDF) [file pcbi.1014387.s002.pdf]

S2 Text. **Supplementary figures**

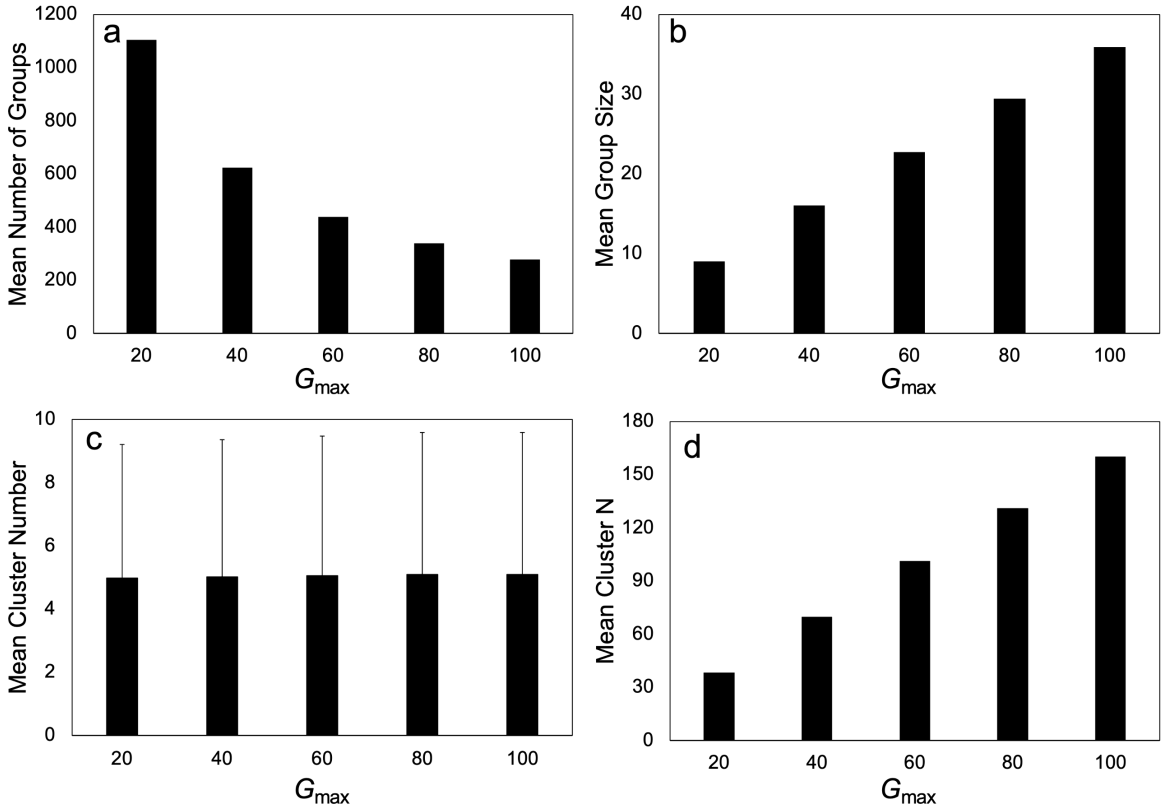

Fig A. Population structure descriptive statistics for values of G_{max}: mean number of groups (a), mean group size (b), and cluster number with standard deviations (c), and mean number of agents *N* in clusters (d).

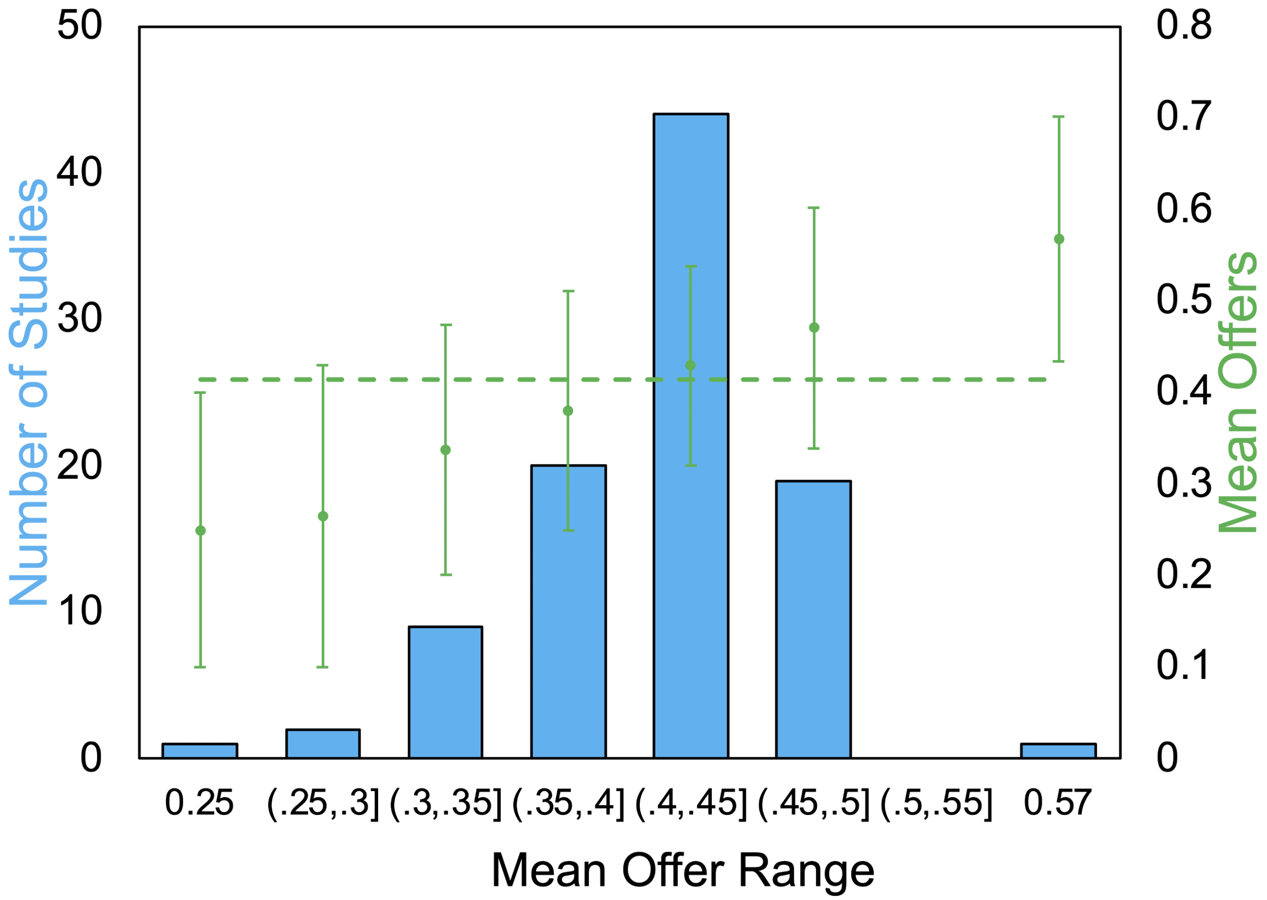

Fig B. Distribution of mean offers across 96 studies. Cochard et al. [1] performed a meta-analysis of 42 papers and a book containing 96 studies on UG experiments conducted in 30 countries from 1983 to 2011. Studies were excluded if they involved: repetition with the same participants; revelation of the mean offer after each round; asymmetric uncertainty; more than two participants or computerized participants; stakes less than $4; earned proposer position; lack of total anonymity; participants having played a game prior to the UG; revelation of the subgame-perfect Nash equilibrium before deciding; or seller/buyer framing. The data are supplementary data from Cochard et al. [1]. Offer means and standard deviations are binned in increments of 5%, the frequency of studies are plotted (blue columns), with mean offers (solid green circles) and standard deviation bars for each set of binned studies. The green dashed horizontal line is the overall mean over the 96 studies (41.5%).

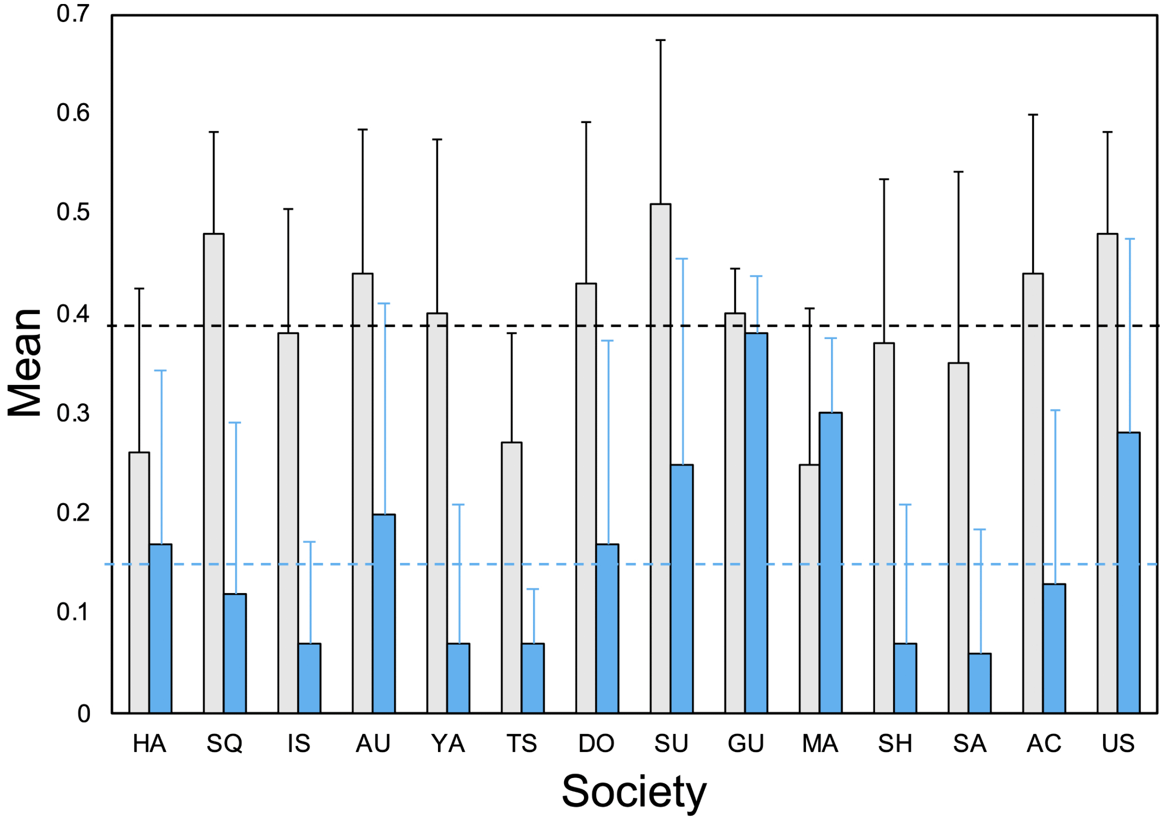

Fig C. Mean offers (gray columns) and ATs (blue columns) with standard deviations across 14 societies from Henrich et al. [2]. The dashed black line is the mean offer across societies, and the dashed blue line is the mean AT across societies. The overall offer-AT gap is 0.23. UG's were conducted in the following societies: Hadza (HA), Sanquianga (SQ), Isanga (IS), Au (AU), Yasawa (YA), Tsimane (TS), Dolgan/Nganasan (DO), Sursurunga (SU), Gusii (GU), Maragoli (MA), Shuar (SH), Samburu (SA), Accra (AC), and U.S./Rural Missouri (US). These data were taken from Table S4 in the Supporting Online Material in Henrich et al. [2].

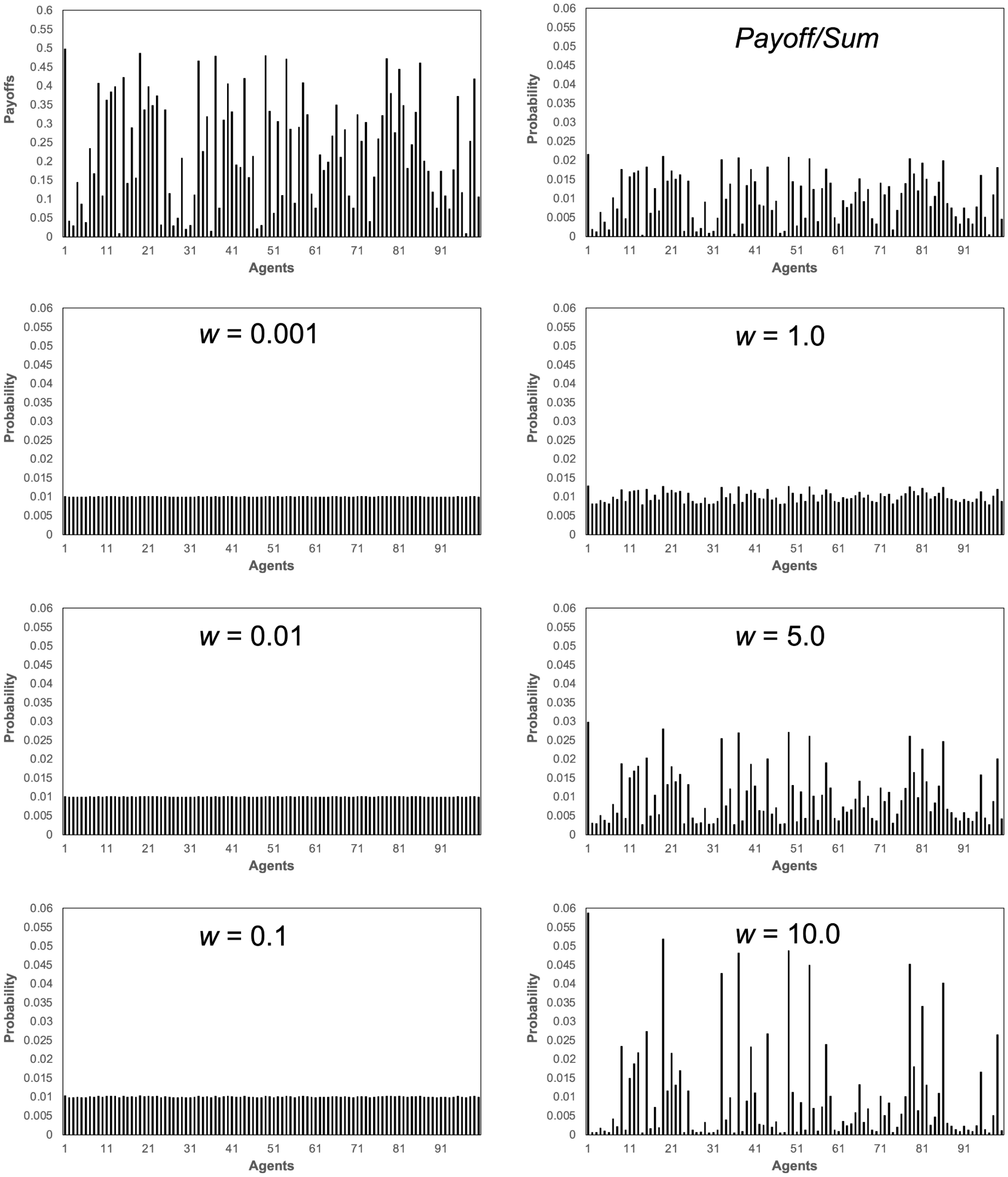

Fig D. Normalized exponential, exp($w\pi_i$), probability distributions for different selection intensities calculated from the top-left payoff distribution. The payoff distribution was generated by drawing random values from a uniform distribution in the range [0, 0.5]. The top-right probability distribution is the payoff distribution divided by its sum. The remainder of the distributions are Normalized exponential probability distributions of the payoff distribution with different values of fitness, *w*.

References

1. Cochard F, Le Gallo J, Georgantzis N, Tisserand JC. Social preferences across different populations: meta-analyses on the ultimatum game and dictator game. J Behav Exp Econ. 2021; 90: 101613. doi:10.1016/j.socec.2020.101613
2. Henrich J, Ensminger J, McElreath R, Barr A, Barrett C, Bolyanatz A, et al. Market, religion, community size and the evolution of fairness and punishment. Science. 2010; 327: 1480–1484. doi:10.1126/science.1182238
