# Supplementary material for: Evolution and the ultimatum game: An agent-based model with interbirth intervals and population structure
Source: PLoS Comput Biol. 2026 Jun 16;22(6):e1014387. doi: 10.1371/journal.pcbi.1014387 (PMC13289883; doi:10.1371/journal.pcbi.1014387)
Supplement: Attachment — Submitted filename: Response to Reviewers.pdf [file pcbi.1014387.s006.pdf]

UCDAVIS
COLLEGE OF LETTERS AND SCIENCE

Department of Psychology

May 31, 2026

Dear PLOS Computational Biology Editor and Reviewers:

In addition to our responses to the editor and reviewers, we made the following changes. First, the figures in the online material S2_Text were converted to TIFF format, and otherwise this file is unchanged from the previous version. Second, as mentioned below, the figure caption for Figure 14 was rearranged for clarity, and so a new version of the manuscript was uploaded with only the Figure 14 caption change described below.

Below are our responses to the editor and reviewers.

Reviewer #2: Thank you for the additional analyses and discussion regarding resource shortages and surpluses. The new simulations help clarify the role of rejection costs and resource constraints in shaping the evolutionary dynamics.

Overall, this revision substantially improves the manuscript. I appreciate that the authors have incorporated my previous suggestions regarding the visual presentations. However, I have noticed a few remaining minor issues that need to be addressed before publication:

Figure 9: The y-axis titles for panel (f) and panel (g) are reversed and need to be corrected.

The y-axis titles are now fixed for panels (f) and (g).

Figure 5: I reiterate my previous recommendation to clearly label the y-axis titles for panel (c) and panel (e). They should explicitly read "Offer Frequency" and "AT Frequency," respectively, to avoid any ambiguity.

The y-axis titles for panels (c) and (e) are now "Offer Frequency" and "AT Frequency," respectively.

Image Quality: Most importantly, the resolution of the currently submitted figures is insufficient. Please ensure that high-resolution, clear image files are provided for all figures in the final production version.

All figures were run through PLOS's NAAS tool. To pass this tool and to make some of the figures more consistent in format, minor changes were made in the following figures:

Tel: 530-752-1880
One Shields Avenue, Davis, CA 95616
pscychology.ucdavis.edu

Figure 6: The figure legend was moved from panel (a) to the bottom of the figure. This allowed the figure to pass the proportionality requirement. For consistency of formatting, G_{max} *row headings were moved to the right side of the figure and formatted as "*G_{max} *= [value]"*
Figure 7: For consistency of formatting, G_{max} *row headings were moved to the right side of the figure as in Figure 6.*

Figure 8: The x-axis and y-axis titles were unbolded.

Figure 11: For consistency, the panel (b) y-axis title was changed to "Offer Frequency" and the panel (c) y-axis title was changed to "AT Frequency".

Figure 14: For clarity, a legend was added to panel (a). In the figure caption, the second sentence, starting with "Results for…," was moved to the end of the caption and referenced the panels it covers.

Figure 15: For consistency of formatting, G_{max} *row headings were moved to the right side of the figure as in Figure 6.*

Figure 16: For consistency of formatting, G_{max} *row headings were moved to the right side of the figure as in Figure 6.*

Sincerely,

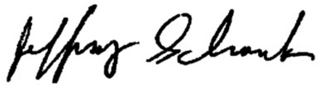

Jeffrey Schank
Department of Psychology
University of California, Davis,
Davis, CA, United States
