# Supplementary material for: Evolution and the ultimatum game: An agent-based model with interbirth intervals and population structure
Source: PLoS Comput Biol. 2026 Jun 16;22(6):e1014387. doi: 10.1371/journal.pcbi.1014387 (PMC13289883; doi:10.1371/journal.pcbi.1014387)
Supplement: Attachment — Submitted filename: Comments_PLOS.docx [file pcbi.1014387.s003.docx]

Reviewer Comments on “Evolution and the ultimatum game: an agent-based model with interbirth intervals and population structure”

The manuscript examines the conditions under which evolutionary processes can give rise to generous offers and offer-acceptance threshold (AT) gaps that resemble those observed in empirical studies of the ultimatum game. The authors develop an innovative agent-based model with biologically realistic constraints, including group structured populations, resource dependent reproduction, and interbirth intervals (IBIs).

This study makes a meaningful contribution by addressing a critical gap in the literature. Previous evolutionary models have recently faced criticism for lacking biological realism and failing to adequately explain empirical data. In contrast, the model presented here is inspired by hunter-gatherer ancestors and utilizes a biologically grounded selection process. The study demonstrates that the synergy between group structure and IBIs provides a robust explanation for observed human behaviors.

A notable strength of the model is its comprehensive scope. Unlike many previous approaches that focus primarily on mean offers, this model accounts for multiple empirical patterns simultaneously, including distributions of offers, AT distributions, and rejection rates. Furthermore, the finding that IBIs are decisive in group-structured populations but negligible in well-mixed ones provides valuable insight into spatial game theory.

**Major concerns**

My main concern is the parameterization of the quantity of resources required to reproduce ($\theta$). There is a contradiction regarding the variable $\theta$ in the current manuscript. Line 230 describes $\theta$ as the "expected accumulation of resources," while Table 2 defines it as "the quantity of resources required to reproduce" with a value of $m\mu$.

If $\theta=m\mu$, an average agent cannot mathematically reach this threshold within time $m$ because the expected payoff per agent is typically $m\mu/2$. This suggests an agent would need at least $2m$ time steps to reproduce. Even if $\theta$ were intended to equal the expected payoff $m\mu/2$, a single rejection would prevent reproduction within the minimum time. Therefore, resource accumulation appears to be the dominant constraint, rather than the IBIs. To verify that the reported synergistic effect is driven by IBIs rather than by a high reproduction threshold, a sensitivity analysis with lower $\theta$ values would be helpful.

**Minor issues**

1. Abstract, Line 44: The word "only" may be too strong ("...only in group-structured populations..."), as it could imply that no other mechanisms can produce these results. It might be better to soften this wording to avoid overstating the necessity of the identified conditions.
2. Table 2: Some symbols appear misaligned with their values and interpretations. This formatting could be checked and adjusted.
3. Line 97: There seems to be an unintended line break here.
4. Line 383: "offer-AT gars" should be "offer-AT gaps."
5. Line 396: The text states results are averaged over $G_{max}$, but Fig 8 presents results separated by $G_{max}$. Clarification here would be helpful.
6. Line 426: The term "rejection rates" may not be accurate in this context. It seems possible that "acceptance thresholds" was intended.
7. Line 462: $G_{max}$ should likely be $N_{max}$.
8. The discussion of offer costs and rejection costs is interesting. It may help readers if the meaning of "rejection costs" is clarified, specifically whether it refers to losses for the pair, the proposer, or the responder.
9. Fig 1: Readability might improve if panels b and c used more specific y axis labels, such as Offer Frequency and AT Frequency.
10. Fig 2 & Fig 3: The population shown in the dashed region of Fig 3 does not visually match Fig 2. If Fig 2 is intended as a zoomed view, it may help to ensure that the visual elements are consistent.
11. Fig 4 & Fig 8: Adding headers such as "With IBIs" and "Without IBIs" could make the figure easier to interpret.
12. Fig 5: It may be helpful to label the y axes of panels c and e more specifically. In addition, panel g appears to be missing the symbol m in the legend.
13. Fig 6: it might be useful to label $G_{max}$ values on the left side, as in Figure 7.
14. Fig 7: The subplot lettering appears inconsistent with both the caption and the references in the main text. This could be checked and aligned, and the subplot columns might also be adjusted for alignment.
15. Fig 9: It might improve clarity to distinguish the y axis titles for the offer and AT panels. Please remove the stray vertical blue line on the far left.
