# Supplementary material for: Evolution and the ultimatum game: An agent-based model with interbirth intervals and population structure
Source: PLoS Comput Biol. 2026 Jun 16;22(6):e1014387. doi: 10.1371/journal.pcbi.1014387 (PMC13289883; doi:10.1371/journal.pcbi.1014387)
Supplement: Attachment — Submitted filename: Response to Reviewers.docx [file pcbi.1014387.s005.docx]

May 2, 2026

Dear PLOS Computational Biology Editor and Reviewers:

We are resubmitting a revised version of “Evolution and the ultimatum game: an agent-based model with interbirth intervals and population structure” for consideration by PLOS Computational Biology.

Below are our responses to the editor and reviewers.

**Additional Editor Comments:**

Both reviewers find the paper to be interesting and generally to be suitable for PLoS Computational Biology.
However, both of them make very good suggestions that I would like the authors to address (e.g., reviewer 1 points out that behavior in the ultimatum game may not necessarily be inherited but also socially learnt. Do the authors have something to say about that alternative possibility?)

*We agree with reviewer 1 that there are alternative possibilities and that it is important to pursue these possibilities. We have discussed some of these possibilities and the challenges they face. We have also added a brief conclusion section to tie the main points together.*

**Reviewers' comments:**

Reviewer's Responses to Questions

**Comments to the Authors:
Please note here if the review is uploaded as an attachment.**

Reviewer #1: The paper presents an agent-based model of the Ultimatum game (UG) that incorporates interbirth intervals and group-structure to explain the evolution of generous offers and gaps in offer-acceptance. They argue that previous evolutionaryu models lack biological realism and that their model better aligns with empirical UG data across societies.

Overall, this is a very well-structured and methodologically detailed paper with a strong theoretical foundation. The simulation design is comprehensive and robust, as are the results, and they align well with meta-analytical UG data.

My main concern stems from the discussion of the limitations of mutation-selection models. I am not convinced that the biologically restricted constraints imposed by this model would provide qualitatively different results from a similar model that includes population structure and/or limited visibility of the strategy space of others in the population. Something similar could be achieved using (for example) a complex network that does not govern interactions, only imitation. I believe the greatest limitation of this paper are perhaps the slightly bold claims associated with their chosen method of selection and interbirth intervals.

*We completely agree about population structure. As you point out, there are a variety of ways of limiting the strategy space in which agents can interact. We chose a simple group structure as roughly inspired by hunter-gatherer populations, but even their structure is more complex. We have added a discussion on this point.*

Otherwise, some small concerns are as follows:

In equation 3, the update rule for resources U_i, t is unclear to me -- does it apply only after reproduction? Please clarify.

*Equation 3 applies when a reproductive event is triggered, though an offspring may not “survive” due to the Moran-like constraint placed on reproduction. We have added a parenthetical remark to help clarify this.*

Minor, but in equation 1 rho is introduced, yet only defined later in Table 2.

*Parenthetical definition added.*

Some parameters seem arbitrary (delta, d and g), the authors should justify these choices or cite relevant literature. Same for m=150 as a 3-year IBI, this is plausbile but not explicitly justified.
The steps chosen for p and q in intervals of 0.1 are a bit puzzling, I would argue that even going up to 0.5 for offers/responses is sufficient, and a finer resolution for p and q, the most important parameters of the model would be achieved.

Perhaps important - the rule for unpaired agents ("randomly chosen partner always keeps nothing") may artificially alter the evolution of responses. Sensitivity analysis for this rule is missing.

*Yes, it does alter the results compared to several other possible ways of handling an odd agent when agents play only once per time step. Because it turns out to be interesting that the three alternatives diverge most from the half-game rule for Nmax = 20, and then converge on the half-game rule for Nmax > 20, we included a new section on these simulations.*

Another minor point that some references are truncated or inconsistently formatted, some author names might be incomplete or some references are cut off.

*Fixed.*

As stated previously, I believe the key issue with the manuscript as it stands is that the discussion is somewhat missing on the importance of cultural evolution, in the sense that UG behaviour can just as well be influenced by cultural transmission and social learning; i.e. population dynamics, social learning, imitation, however one might want to name this. Rather than overstate the generability of the chosen (perhaps more biologically plausible?) method, it would be useful to see how a similar model could be generated with very well established imitation dynamics (i.e. Moran processes, Fermi update, etc.), perhaps even tested against a similar model for robustness. Personally, I don't see how a similar model would not be achievable using population structures, yielding possibly very similar results that also align with empirical data. At the very least, I would like the discussion to highlight this.

*We have added substantial discussion of social learning as imitation, the challenge of developing such models, and population structure.*

Many thanks for the very well written manuscript.

**Reviewer #2**: Please find my detailed comments and suggestions in the uploaded attachment.

Reviewer Comments on “Evolution and the ultimatum game: an agent-based model with interbirth intervals and population structure”

The manuscript examines the conditions under which evolutionary processes can give rise to generous offers and offer-acceptance threshold (AT) gaps that resemble those observed in empirical studies of the ultimatum game. The authors develop an innovative agent-based model with biologically realistic constraints, including group structured populations, resource dependent reproduction, and interbirth intervals (IBIs).

This study makes a meaningful contribution by addressing a critical gap in the literature. Previous evolutionary models have recently faced criticism for lacking biological realism and failing to adequately explain empirical data. In contrast, the model presented here is inspired by hunter-gatherer ancestors and utilizes a biologically grounded selection process. The study demonstrates that the synergy between group structure and IBIs provides a robust explanation for observed human behaviors.

A notable strength of the model is its comprehensive scope. Unlike many previous approaches that focus primarily on mean offers, this model accounts for multiple empirical patterns simultaneously, including distributions of offers, AT distributions, and rejection rates. Furthermore, the finding that IBIs are decisive in group-structured populations but negligible in well-mixed ones provides valuable insight into spatial game theory.

**Major concerns**

My main concern is the parameterization of the quantity of resources required to reproduce ($\theta$). There is a contradiction regarding the variable $\theta$ in the current manuscript. Line 230 describes $\theta$ as the "expected accumulation of resources," while Table 2 defines it as "the quantity of resources required to reproduce" with a value of $m\mu$. If $\theta=m\mu$, an average agent cannot mathematically reach this threshold within time $m$ because the expected payoff per agent is typically $m\mu/2$. This suggests an agent would need at least $2m$ time steps to reproduce.

*This was an embarrassing mistake in Table 2 that we simply missed. Indeed,* *θ = mμ/2 = 5m, as stated in the text and the simulations.*

Even if $\theta$ were intended to equal the expected payoff $m\mu/2$, a single rejection would prevent reproduction within the minimum time. Therefore, resource accumulation appears to be the dominant constraint, rather than the IBIs. To verify that the reported synergistic effect is driven by IBIs rather than by a high reproduction threshold, a sensitivity analysis with lower $\theta$ values would be helpful.

*It is important to keep in mind that the exact same thresholds were used in the simulations without IBIs, thereby providing theoretical control conditions for the IBIs. In addition, in Fig. 7, we drew the expected resources from a truncated normal distribution with a standard deviation up to 0.75θ = 7.5, which would generate considerable inter-agent variation in resource accumulation. For this revision, we also added a new set of simulations in which θ was systematically decreased by 10%, resulting in resource surpluses, and increased by 10%, resulting in resource shortages. For resource shortages, the evolved mean offers and ATs (Fig. 15 a-d) decreased as θ increased. For lower values of θ, the results were much more complex (Fig. 15 e-h), with interactions involving G_max_ and m, which we discuss in the discussion section on “Offer and rejection costs.”*

**Minor issues**

1. Abstract, Line 44: The word "only" may be too strong ("...only in group-structured populations..."), as it could imply that no other mechanisms can produce these results. It might be better to soften this wording to avoid overstating the necessity of the identified conditions.

*The “only” was intended to refer to the current model. To clarify, the sentence now begins: “For the proposed model…”*

2. Table 2: Some symbols appear misaligned with their values and interpretations. This formatting could be checked and adjusted.

*Better aligned now. Word appears to alter line space when characters are subscripted.*

3. Line 97: There seems to be an unintended line break here.

*Fixed*

4. Line 383: "offer-AT gars" should be "offer-AT gaps."

*Fixed*

5. Line 396: The text states results are averaged over $G_{max}$, but Fig 8 presents results separated by $G_{max}$. Clarification here would be helpful.

*We left out a few clarifying words, which have not been added.*

6. Line 426: The term "rejection rates" may not be accurate in this context. It seems possible that "acceptance thresholds" was intended.

*Correct and fixed.*

7. Line 462: $G_{max}$ should likely be $N_{max}$.

*Fixed.*

8. The discussion of offer costs and rejection costs is interesting. It may help readers if the meaning of "rejection costs" is clarified, specifically whether it refers to losses for the pair, the proposer, or the responder.

*We have clarified that rejection costs apply to both the proposer and the responder and can have large systematic effects at the group level.*

9. Fig 1: Readability might improve if panels b and c used more specific y axis labels, such as Offer Frequency and AT Frequency.

*Done.*

10. Fig 2 & Fig 3: The population shown in the dashed region of Fig 3 does not visually match Fig 2. If Fig 2 is intended as a zoomed view, it may help to ensure that the visual elements are consistent.

*Fixed*

11. Fig 4 & Fig 8: Adding headers such as "With IBIs" and "Without IBIs" could make the figure easier to interpret.

*Done*

12. Fig 5: It may be helpful to label the y axes of panels c and e more specifically. In addition, panel g appears to be missing the symbol m in the legend.

*Done*

13. Fig 6: it might be useful to label $G_{max}$ values on the left side, as in Figure 7.

*Done*

14. Fig 7: The subplot lettering appears inconsistent with both the caption and the references in the main text. This could be checked and aligned, and the subplot columns might also be adjusted for alignment.

*Done*

15. Fig 9: It might improve clarity to distinguish the y axis titles for the offer and AT panels. Please remove the stray vertical blue line on the far left.

*Done*

Sincerely,

| Jeffrey Schank  Department of Psychology  University of California, Davis,  Davis, CA, United States |  |
| --- | --- |
