# Supplementary material for: Evolution and the ultimatum game: An agent-based model with interbirth intervals and population structure
Source: PLoS Comput Biol. 2026 Jun 16;22(6):e1014387. doi: 10.1371/journal.pcbi.1014387 (PMC13289883; doi:10.1371/journal.pcbi.1014387)
Supplement: Peer review history

# Peer review: Decision Letter 0

**DOI:** 10.1371/journal.pcbi.1014387.r001

**Authors:** Zhaolei Zhang, Christian Hilbe

24 Feb 2026

PCOMPBIOL-D-25-02626

Evolution and the ultimatum game: an agent-based model with interbirth intervals and population structure

PLOS Computational Biology

Dear Dr. Schank,

Thank you for submitting your manuscript to PLOS Computational Biology. After careful consideration, we feel that it has merit but does not fully meet PLOS Computational Biology's publication criteria as it currently stands. Therefore, we invite you to submit a revised version of the manuscript that addresses the points raised during the review process.

Please submit your revised manuscript by Apr 26 2026 11:59PM. If you will need more time than this to complete your revisions, please reply to this message or contact the journal office at ploscompbiol@plos.org. When you're ready to submit your revision, log on to https://www.editorialmanager.com/pcompbiol/ and select the 'Submissions Needing Revision' folder to locate your manuscript file.

Please include the following items when submitting your revised manuscript:

* A letter that responds to each point raised by the editor and reviewer(s). You should upload this letter as a separate file labeled 'Response to Reviewers'. This file does not need to include responses to formatting updates and technical items listed in the 'Journal Requirements' section below.

* A marked-up copy of your manuscript that highlights changes made to the original version. You should upload this as a separate file labeled 'Revised Manuscript with Track Changes'.

* An unmarked version of your revised paper without tracked changes. You should upload this as a separate file labeled 'Manuscript'.

If you would like to make changes to your financial disclosure, competing interests statement, or data availability statement, please make these updates within the submission form at the time of resubmission. Guidelines for resubmitting your figure files are available below the reviewer comments at the end of this letter.

We look forward to receiving your revised manuscript.

Kind regards,

Christian Hilbe

Academic Editor

PLOS Computational Biology

Zhaolei Zhang

Section Editor

PLOS Computational Biology

**Additional Editor Comments:**

Both reviewers find the paper to be interesting and generally to be suitable for PLoS Computational Biology.

However, both of them make very good suggestions that I would like the authors to address (e.g., reviewer 1 points out that behavior in the ultimatum game may not necessarily be inherited but also socially learnt. Do the authors have something to say about that alternative possibility?)

**Journal Requirements:**

1) Please ensure that the CRediT author contributions listed for every co-author are completed accurately and in full.

At this stage, the following Authors/Authors require contributions: Jeffrey C. Schank, and Matt L. Miller. Please ensure that the full contributions of each author are acknowledged in the "Add/Edit/Remove Authors" section of our submission form.

The list of CRediT author contributions may be found here: https://journals.plos.org/ploscompbiol/s/authorship#loc-author-contributions

2) We ask that a manuscript source file is provided at Revision. Please upload your manuscript file as a .doc, .docx, .rtf or .tex. If you are providing a .tex file, please upload it under the item type u2018LaTeX Source Fileu2019 and leave your .pdf version as the item type u2018Manuscriptu2019.

3) Please upload all main figures as separate Figure files in .tif or .eps format. For more information about how to convert and format your figure files please see our guidelines:

https://journals.plos.org/ploscompbiol/s/figures

**Reviewers' comments:**

Reviewer's Responses to Questions

**Comments to the Authors:**

**Please note here if the review is uploaded as an attachment.**

Reviewer #1: The paper presents an agent-based model of the Ultimatum game (UG) that incorporates interbirth intervals and group-structure to explain the evolution of generous offers and gaps in offer-acceptance. They argue that previous evolutionaryu models lack biological realism and that their model better aligns with empirical UG data across societies.

Overall, this is a very well-structured and methodologically detailed paper with a strong theoretical foundation. The simulation design is comprehensive and robust, as are the results, and they align well with meta-analytical UG data.

My main concern stems from the discussion of the limitations of mutation-selection models. I am not convinced that the biologically restricted constraints imposed by this model would provide qualitatively different results from a similar model that includes population structure and/or limited visibility of the strategy space of others in the population. Something similar could be achieved using (for example) a complex network that does not govern interactions, only imitation. I believe the greatest limitation of this paper are perhaps the slightly bold claims associated with their chosen method of selection and interbirth intervals.

Otherwise, some small concerns are as follows:

In equation 3, the update rule for resources U_i, t is unclear to me -- does it apply only after reproduction? Please clarify.

Minor, but in equation 1 rho is introduced, yet only defined later in Table 2.

Some parameters seem arbitrary (delta, d and g), the authors should justify these choices or cite relevant literature. Same for m=150 as a 3-year IBI, this is plausbile but not explicitly justified.

The steps chosen for p and q in intervals of 0.1 are a bit puzzling, I would argue that even going up to 0.5 for offers/responses is sufficient, and a finer resolution for p and q, the most important parameters of the model would be achieved.

Perhaps important - the rule for unpaired agents ("randomly chosen partner always keeps nothing") may artificially alter the evolution of responses. Sensitivity analysis for this rule is missing.

Another minor point that some references are truncated or inconsistently formatted, some author names might be incomplete or some references are cut off.

As stated previously, I believe the key issue with the manuscript as it stands is that the discussion is somewhat missing on the importance of cultural evolution, in the sense that UG behaviour can just as well be influenced by cultural transmission and social learning; i.e. population dynamics, social learning, imitation, however one might want to name this. Rather than overstate the generability of the chosen (perhaps more biologically plausible?) method, it would be useful to see how a similar model could be generated with very well established imitation dynamics (i.e. Moran processes, Fermi update, etc.), perhaps even tested against a similar model for robustness. Personally, I don't see how a similar model would not be achievable using population structures, yielding possibly very similar results that also align with empirical data. At the very least, I would like the discussion to highlight this.

Many thanks for the very well written manuscript.

Reviewer #2: Please find my detailed comments and suggestions in the uploaded attachment.

**********

**Have the authors made all data and (if applicable) computational code underlying the findings in their manuscript fully available?**

The PLOS Data policy requires authors to make all data and code underlying the findings described in their manuscript fully available without restriction, with rare exception (please refer to the Data Availability Statement in the manuscript PDF file). The data and code should be provided as part of the manuscript or its supporting information, or deposited to a public repository. For example, in addition to summary statistics, the data points behind means, medians and variance measures should be available. If there are restrictions on publicly sharing data or code —e.g. participant privacy or use of data from a third party—those must be specified.

Reviewer #1: Yes

Reviewer #2: Yes

**********

PLOS authors have the option to publish the peer review history of their article (what does this mean?). If published, this will include your full peer review and any attached files.

If you choose “no”, your identity will remain anonymous but your review may still be made public.

**Do you want your identity to be public for this peer review?**  For information about this choice, including consent withdrawal, please see our Privacy Policy.

Reviewer #1: **Yes:** Theodor Cimpeanu

Reviewer #2: No

[NOTE: If reviewer comments were submitted as an attachment file, they will be attached to this email and accessible via the submission site. Please log into your account, locate the manuscript record, and check for the action link "View Attachments". If this link does not appear, there are no attachment files.]

**Figure resubmission:**

-->While revising your submission, we strongly recommend that you use PLOS’s NAAS tool (https://ngplosjournals.pagemajik.ai/artanalysis) to test your figure files. NAAS can convert your figure files to the TIFF file type and meet basic requirements (such as print size, resolution), or provide you with a report on issues that do not meet our requirements and that NAAS cannot fix.

After uploading your figures to PLOS’s NAAS tool - https://ngplosjournals.pagemajik.ai/artanalysis, NAAS will process the files provided and display the results in the "Uploaded Files" section of the page as the processing is complete. If the uploaded figures meet our requirements (or NAAS is able to fix the files to meet our requirements), the figure will be marked as "fixed" above. If NAAS is unable to fix the files, a red "failed" label will appear above. When NAAS has confirmed that the figure files meet our requirements, please download the file via the download option, and include these NAAS processed figure files when submitting your revised manuscript.

**Reproducibility:**

To enhance the reproducibility of your results, we recommend that authors of applicable studies deposit laboratory protocols in protocols.io, where a protocol can be assigned its own identifier (DOI) such that it can be cited independently in the future. Additionally, PLOS ONE offers an option to publish peer-reviewed clinical study protocols. Read more information on sharing protocols at https://plos.org/protocols?utm_medium=editorial-email&utm_source=authorletters&utm_campaign=protocols

Attachment Submitted filename: Comments_PLOS.docx

# Author comment: Author response to Decision Letter 1

**DOI:** 10.1371/journal.pcbi.1014387.r002

4 May 2026

Attachment Submitted filename: Response to Reviewers.docx

# Peer review: Decision Letter 1

**DOI:** 10.1371/journal.pcbi.1014387.r003

**Authors:** Zhaolei Zhang, Christian Hilbe, Zhaolei Zhang, Christian Hilbe

22 May 2026

PCOMPBIOL-D-25-02626R1

Evolution and the ultimatum game: an agent-based model with interbirth intervals and population structure

PLOS Computational Biology

Dear Dr. Schank,

Thank you for submitting your manuscript to PLOS Computational Biology. After careful consideration, we feel that it has merit but does not fully meet PLOS Computational Biology's publication criteria as it currently stands. Therefore, we invite you to submit a revised version of the manuscript that addresses the points raised during the review process.

Please submit your revised manuscript by Jul 22 2026 11:59PM. If you will need more time than this to complete your revisions, please reply to this message or contact the journal office at ploscompbiol@plos.org. When you're ready to submit your revision, log on to https://www.editorialmanager.com/pcompbiol/ and select the 'Submissions Needing Revision' folder to locate your manuscript file.

Please include the following items when submitting your revised manuscript:

* A letter that responds to each point raised by the editor and reviewer(s). You should upload this letter as a separate file labeled 'Response to Reviewers'. This file does not need to include responses to formatting updates and technical items listed in the 'Journal Requirements' section below.

* A marked-up copy of your manuscript that highlights changes made to the original version. You should upload this as a separate file labeled 'Revised Manuscript with Track Changes'.

* An unmarked version of your revised paper without tracked changes. You should upload this as a separate file labeled 'Manuscript'.

If you would like to make changes to your financial disclosure, competing interests statement, or data availability statement, please make these updates within the submission form at the time of resubmission. Guidelines for resubmitting your figure files are available below the reviewer comments at the end of this letter.

As the corresponding author, your ORCID iD is verified in the submission system and will appear in the published article. PLOS supports the use of ORCID, and we encourage all coauthors to register for an ORCID iD and use it as well. Please encourage your coauthors to verify their ORCID iD within the submission system before final acceptance, as unverified ORCID iDs will not appear in the published article. Only the individual author can complete the verification step; PLOS staff cannot verify ORCID iDs on behalf of authors.

We look forward to receiving your revised manuscript.

Kind regards,

Christian Hilbe

Academic Editor

PLOS Computational Biology

Zhaolei Zhang

Section Editor

PLOS Computational Biology

**Additional Editor Comments:**

The manuscript has been re-sent to the original two reviewers, and they find the paper to be greatly improved.

Reviewer 2 correctly noted that the figure quality in the pdf is poor; I did see that the authors had uploaded figures with higher solution, but those figures require require considerable memory space, so they probably got compressed.

I would like to encourage the authors to address the remaining comments of reviewer 2, and to re-upload the figures (if possible) in a vector-based format (e.g. eps instead of tif).

**Reviewers' comments:**

Reviewer's Responses to Questions

**Comments to the Authors:**

**Please note here if the review is uploaded as an attachment.**

Reviewer #1: My concerns have been addressed and I am happy to recommend this for acceptance. Thank you very much to the authors for valuing the reviewers' feedback.

Reviewer #2: Thank you for the additional analyses and discussion regarding resource shortages and surpluses. The new simulations help clarify the role of rejection costs and resource constraints in shaping the evolutionary dynamics.

Overall, this revision substantially improves the manuscript. I appreciate that the authors have incorporated my previous suggestions regarding the visual presentations. However, I have noticed a few remaining minor issues that need to be addressed before publication:

Figure 9: The y-axis titles for panel (f) and panel (g) are reversed and need to be corrected.

Figure 5: I reiterate my previous recommendation to clearly label the y-axis titles for panel (c) and panel (e). They should explicitly read "Offer Frequency" and "AT Frequency," respectively, to avoid any ambiguity.

Image Quality: Most importantly, the resolution of the currently submitted figures is insufficient. Please ensure that high-resolution, clear image files are provided for all figures in the final production version.

**********

**Have the authors made all data and (if applicable) computational code underlying the findings in their manuscript fully available?**

The PLOS Data policy requires authors to make all data and code underlying the findings described in their manuscript fully available without restriction, with rare exception (please refer to the Data Availability Statement in the manuscript PDF file). The data and code should be provided as part of the manuscript or its supporting information, or deposited to a public repository. For example, in addition to summary statistics, the data points behind means, medians and variance measures should be available. If there are restrictions on publicly sharing data or code —e.g. participant privacy or use of data from a third party—those must be specified.

Reviewer #1: None

Reviewer #2: None

**********

PLOS authors have the option to publish the peer review history of their article (what does this mean?). If published, this will include your full peer review and any attached files.

If you choose “no”, your identity will remain anonymous but your review may still be made public.

**Do you want your identity to be public for this peer review?**  For information about this choice, including consent withdrawal, please see our Privacy Policy.

Reviewer #1: No

Reviewer #2: No

[NOTE: If reviewer comments were submitted as an attachment file, they will be attached to this email and accessible via the submission site. Please log into your account, locate the manuscript record, and check for the action link "View Attachments". If this link does not appear, there are no attachment files.]

**Figure resubmission:**

-->While revising your submission, we strongly recommend that you use PLOS’s NAAS tool (https://ngplosjournals.pagemajik.ai/artanalysis) to test your figure files. NAAS can convert your figure files to the TIFF file type and meet basic requirements (such as print size, resolution), or provide you with a report on issues that do not meet our requirements and that NAAS cannot fix.

After uploading your figures to PLOS’s NAAS tool - https://ngplosjournals.pagemajik.ai/artanalysis, NAAS will process the files provided and display the results in the "Uploaded Files" section of the page as the processing is complete. If the uploaded figures meet our requirements (or NAAS is able to fix the files to meet our requirements), the figure will be marked as "fixed" above. If NAAS is unable to fix the files, a red "failed" label will appear above. When NAAS has confirmed that the figure files meet our requirements, please download the file via the download option, and include these NAAS processed figure files when submitting your revised manuscript.

**Reproducibility:**

To enhance the reproducibility of your results, we recommend that authors of applicable studies deposit laboratory protocols in protocols.io, where a protocol can be assigned its own identifier (DOI) such that it can be cited independently in the future. Additionally, PLOS ONE offers an option to publish peer-reviewed clinical study protocols. Read more information on sharing protocols at https://plos.org/protocols?utm_medium=editorial-email&utm_source=authorletters&utm_campaign=protocols

# Author comment: Author response to Decision Letter 2

**DOI:** 10.1371/journal.pcbi.1014387.r004

31 May 2026

Attachment Submitted filename: Response to Reviewers.pdf

# Editor report: Decision Letter 2

**DOI:** 10.1371/journal.pcbi.1014387.r005

**Authors:** Zhaolei Zhang, Christian Hilbe, Zhaolei Zhang, Christian Hilbe, Zhaolei Zhang, Christian Hilbe

1 Jun 2026

Dear Prof. Schank,

We are pleased to inform you that your manuscript 'Evolution and the ultimatum game: an agent-based model with interbirth intervals and population structure' has been provisionally accepted for publication in PLOS Computational Biology.

Before your manuscript can be formally accepted you will need to complete some formatting changes, which you will receive in a follow up email. A member of our team will be in touch with a set of requests.

Please note that your manuscript will not be scheduled for publication until you have made the required changes, so a swift response is appreciated.

IMPORTANT: The editorial review process is now complete. PLOS will only permit corrections to spelling, formatting or significant scientific errors from this point onwards. Requests for major changes, or any which affect the scientific understanding of your work, will cause delays to the publication date of your manuscript.

Should you, your institution's press office or the journal office choose to press release your paper, you will automatically be opted out of early publication. We ask that you notify us now if you or your institution is planning to press release the article. All press must be co-ordinated with PLOS.

Thank you again for supporting Open Access publishing; we are looking forward to publishing your work in PLOS Computational Biology.

Best regards,

Christian Hilbe

Academic Editor

PLOS Computational Biology

Zhaolei Zhang

Section Editor

PLOS Computational Biology

***********************************************************

The author has taken all remaining comments into account.

The figures still look pixelated in the automatically-created pdf version, but I trust the author that they are publication ready.

# Editor report: Acceptance letter

**DOI:** 10.1371/journal.pcbi.1014387.r006

**Authors:** Zhaolei Zhang, Christian Hilbe, Zhaolei Zhang, Christian Hilbe, Zhaolei Zhang, Christian Hilbe

PCOMPBIOL-D-25-02626R2

Evolution and the ultimatum game: an agent-based model with interbirth intervals and population structure

Dear Dr Schank,

I am pleased to inform you that your manuscript has been formally accepted for publication in PLOS Computational Biology. Your manuscript is now with our production department and you will be notified of the publication date in due course.

The corresponding author will soon be receiving a typeset proof for review, to ensure errors have not been introduced during production. Please review the PDF proof of your manuscript carefully, as this is the last chance to correct any errors. Please note that major changes, or those which affect the scientific understanding of the work, will likely cause delays to the publication date of your manuscript.

Soon after your final files are uploaded, unless you have opted out, the early version of your manuscript will be published online. The date of the early version will be your article's publication date. The final article will be published to the same URL, and all versions of the paper will be accessible to readers.

For Research, Software, and Methods articles, you will receive an invoice from PLOS for your publication fee after your manuscript has reached the completed accept phase. If you receive an email requesting payment before acceptance or for any other service, this may be a phishing scheme. Learn how to identify phishing emails and protect your accounts at https://explore.plos.org/phishing.

Thank you again for supporting PLOS Computational Biology and open-access publishing. We are looking forward to publishing your work!

With kind regards,

Anita Estes

PLOS Computational Biology | Carlyle House, Carlyle Road, Cambridge CB4 3DN | United Kingdom ploscompbiol@plos.org | Phone +44 (0) 1223-442824 | ploscompbiol.org | @PLOSCompBiol
